# Thermal Alteration in Adsorption Sites over SAPO‐34 Zeolite

**DOI:** 10.1002/anie.202204500

**Published:** 2022-05-12

**Authors:** Guangchao Li, Tatchamapan Yoskamtorn, Wei Chen, Christopher Foo, Jianwei Zheng, Chiu Tang, Sarah Day, Anmin Zheng, Molly Meng‐Jung Li, Shik Chi Edman Tsang

**Affiliations:** ^1^ Wolfson Catalysis Centre Department of Chemistry University of Oxford Oxford OX1 3QR UK; ^2^ State Key Laboratory of Magnetic Resonance and Atomic and Molecular Physics National Center for Magnetic Resonance in Wuhan Wuhan Institute of Physics and Mathematics Innovation Academy for Precision Measurement Science and Technology Chinese Academy of Sciences Wuhan 430071 China; ^3^ Diamond Light Source Ltd. Didcot OX11 0DE UK; ^4^ Department of Applied Physics The Hong Kong Polytechnic University Hong Kong

**Keywords:** Active Sites, Frustrated Lewis Pair, Synchrotron X-Ray Diffraction, Solid-State Nuclear Magnetic Resonance, Zeolites

## Abstract

Zeolites have found tremendous applications in the chemical industry. However, the dynamic nature of their active sites under the flow of adsorbate molecules for adsorption and catalysis is unclear, especially in operando conditions, which could be different from the as‐synthesized structures. In the present study, we report a structural transformation of the adsorptive active sites in SAPO‐34 zeolite by using acetone as a probe molecule under various temperatures. The combination of solid‐state nuclear magnetic resonance, in situ variable‐temperature synchrotron X‐ray diffraction, and in situ diffuse‐reflectance infrared Fourier‐transform spectroscopy allow a clear identification and quantification that the chemisorption of acetone can convert the classical Brønsted acid site adsorption mode to an induced Frustrated Lewis Pairs adsorption mode at increasing temperatures. Such facile conversion is also supported by the calculations of ab‐initio molecular‐dynamics simulations. This work sheds new light on the importance of the dynamic structural alteration of active sites in zeolites with adsorbates at elevated temperatures.

## Graphical abstract

To better identify the structure of the active sites in zeolites under real reaction conditions, we demonstrate that the adsorption mode of acetone over classical Brønsted acid sites (BAS) can be thermally converted into the induced Frustrated Lewis Pair (FLP) adsorption mode in SAPO‐34 zeolite.

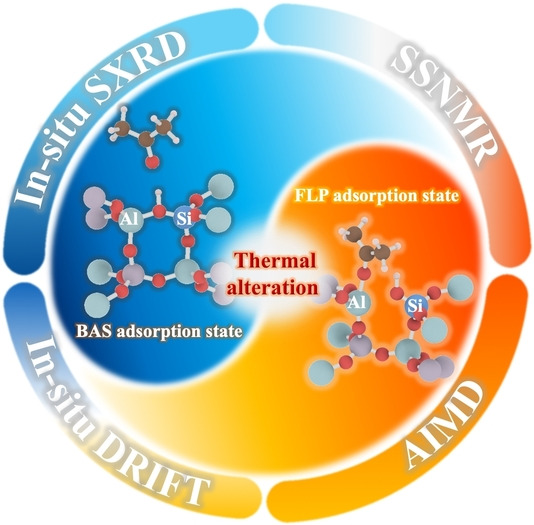

## Introduction

The elucidation of the active sites’ structure in heterogeneous catalysts under real reaction conditions is one of the most important challenges facing the scientific community.^[1]^ There is an increasing amount of evidence by the in situ/operando characterization that active sites can be generated by the interaction of substrate molecules with the catalysts,^[2]^ which can result in the significant promotion of catalytic performance. For example, the initial modification of catalysts under reaction conditions by coke deposition,^[3]^ water dissociation,^[4]^ partial reduction,^[5]^ etc., would greatly affect the catalytic behaviors. On the other hand, surface rearrangement could occur under reaction conditions, which may cause dynamic changes in the active sites.^[6]^ Therefore, the real active sites under working conditions could be significantly different from those characterized under ex situ conditions. Perhaps, it would be easier to assume the dynamic nature of the surface rather than bulk sites plays a critical role in heterogeneous catalysis due to the low barrier for surface reconstruction.[^2^, ^7^] Yet, the in situ identification of the dynamic active sites in zeolites under reaction conditions remains under‐explored, due to the widespread assumption of their structural rigidity/stability during the reactions as well as various limiting issues with characterization techniques.^[8]^

Zeolites, as one of the most important heterogeneous catalysts, have been widely applied in the petrochemical industry. Thus, they are continuously the subject of vast interests from both industry and academia. Over the past few decades, tremendous scientific efforts have been dedicated to the investigation of active sites in zeolites using various new experimental and theoretical techniques, including solid‐state nuclear magnetic resonance (SSNMR),^[9]^ Fourier transform infrared (FT‐IR) spectroscopy,^[10]^ X‐ray absorption spectroscopy (XAS),^[11]^ X‐ray emission spectroscopy (XES),^[12]^ and molecular simulations,^[13]^ etc. Nowadays, there is a growing interest in the study of the “flexible framework” of zeolites which have long been treated as rigid and robust inorganic porous solid materials.^[14]^ The reversible changes of their structures and properties in response to external stimuli have been reported in a large number of works during the past time.[^14f^, ^14g^, ^14h^, ^14i^, ^14j^] In particular, the framework flexibility accounted for the newly created active sites in zeolites has been well recognized. Very recently, Xiao et al.^[7c]^ reported that the hydrolysis of the B−O−Si into B−OH and Si−OH by water in boron‐doped zeolites is operated as the active site for the oxidative dehydrogenation of propane. By using SSNMR and FT‐IR, Ivanova et al.^[15]^ also reported that the Sn−O−Si bond of Sn‐Beta zeolite could be induced to break into Si−OH and Sn‐OH by water adsorption, thus inducing Brønsted acidity. Therefore, the interaction between the adsorbates and the dynamic framework of zeolites could affect the acid properties during catalysis.^[16]^

In general, there are two types of active sites for molecular adsorption and catalysis in zeolites: Brønsted acid site (BAS) and Lewis acid site (LAS). Using the classical rigid model for the framework, the former is assigned to a proton attached to bridging oxygen between substituted Al and Si (Si−O(H)−Al) due to charge balancing. Meanwhile, the latter is the extra‐framework Al species inside the zeolite, which are deposited after their synthesis.^[7a]^ For zeolites without dealumination like SAPO zeolites, the typical adsorption mode of adsorbates should therefore corresponds only to the hydrogen‐bonding interaction with the proton of BAS. Apart from this BAS adsorption mode, we have recently shown that there is an atypical adsorption configuration, which we refer to as the induced Frustrated Lewis pair (FLP) adsorption mode (Figure S1).^[16]^ In the FLP adsorption geometry, the Lewis basic atom of the adsorbate is directly bonded to the Lewis acidic Al site, while the Lewis acid atom of the adsorbate is directly bonded to the Lewis basic SiO(H).^[7a]^ This indicates that the Lewis pair sites from dative bonded Al−O(H)Si in a rigid framework can still be made available for adsorption by the competitive cleavage of the Al−O bond in BAS under exposure to polar adsorbates. We also demonstrated that the polarity of the adsorbate significantly impacts the induced FLP sites. Higher polarity of adsorbates such as water lead to a greater proportion of induced FLP than BAS.

Besides the polarity of adsorbates, temperature could be another key factor that may affect the creation of induced FLP adsorption sites from BAS since structural lability is related to entropic effects. Given that most of zeolite‐catalyzed reactions in petrochemical interests are triggered at high temperatures,^[17]^ thus the active sites under the high temperature/working conditions are significantly different from those often characterized under room temperature using ex situ techniques. For instance, three‐fold coordinated framework aluminum is generated at high temperature in Beta zeolites, as shown by van Bokhoven et al.^[18]^ using in situ X‐ray absorption near‐edge spectroscopy. The temperature‐dependent properties of BAS have also been demonstrated by variable‐temperature SSNMR^[19]^ and infrared experiments.^[20]^ Therefore, the investigation of the temperature effect on the induced structural changes of active sites in the presence of adsorbate molecules is of great significance. Moreover, it could offer further understanding of the dynamic catalysis by zeolites.

Acetone, as a well‐adopted probe molecule, has been widely used to detect the acidity of solid materials.^[21]^ In the present study, we employed acetone molecules to probe the adsorption‐induced structural changes of active sites in SAPO‐34 under different temperatures by SSNMR, in situ diffuse‐reflectance infrared Fourier‐transform (DRIFT) spectroscopy, in situ variable‐temperature synchrotron X‐ray diffraction (VT‐SXRD), and ab‐initio molecular dynamics (AIMD) simulations. We report that our experimental findings combined with theoretical studies provide direct evidence for the dynamic modification of adsorption sites for acetone in this zeolite under various temperatures, which suggests that the real molecular adsorption sites and distribution at different temperatures do not resemble those at room temperature. Thus, this work addresses the urgent need for the elucidation of the dynamic structure of catalytic sites in zeolites, providing the foundation for a more comprehensive understanding of catalytic mechanisms under reaction conditions in the future.

## Results and Discussion

**Figure 1:**
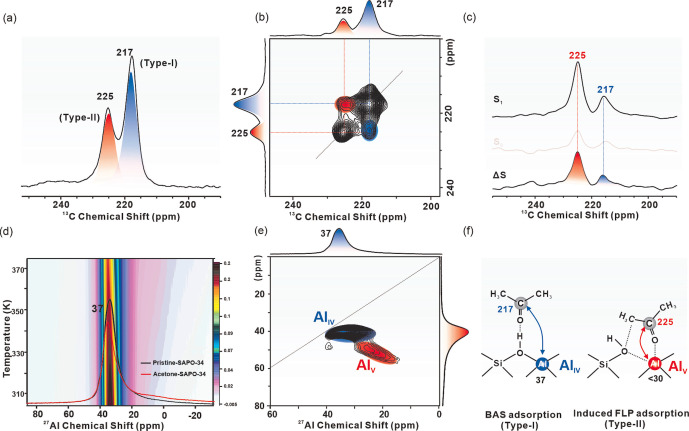
a) ^13^C CPMAS SSNMR of SAPO‐34 adsorbed with ^13^C‐2‐acetone at 298 K, b) 2D ^13^C‐^13^C PDSD MAS SSNMR spectrum of SAPO‐34 adsorbed with ^13^C‐2‐acetone. c) ^13^C‐^27^Al SRESPDOR SSNMR spectra of SAPO‐34 adsorbed with ^13^C‐2‐acetone at 298 K, S_1_ and S_0_ represent the spectra observed with and without ^13^C‐^27^Al SRESPDOR dipolar dephasing (with a recoupling period of *τ*=16 ms), respectively. ΔS represents the spectrum difference of S_1_ and S_0_, d) ^27^Al one pulse MAS SSNMR spectrum of SAPO‐34 before (black) and after (colors) adsorption of ^13^C‐2‐acetone, and the corresponding 2D spectra after separately heating the rotor with the adsorbed sample to 323 K, 348 K, and 373 K, all data were collected under 298 K, e) ^27^Al MQ MAS SSNMR spectrum of SAPO‐34 adsorbed with ^13^C‐2‐acetone at 298 K, f) proposed acetone adsorption modes in SAPO‐34.

Firstly, SSNMR experiments were conducted to investigate the adsorption behaviors of ^13^C‐2‐acetone over SAPO‐34. Results and explanations about the basic characterization of the calcined pristine SAPO‐34, including ^1^H, ^27^Al, ^29^Si, ^31^P Magic Angle Spinning (MAS) SSNMR spectra, are available in Supporting Information (Scheme S1 and Figures S2–S4). Upon adsorption of ^13^C‐2‐acetone, 1D ^13^C cross‐polarization (CP) MAS SSNMR clearly shows that there are two types of ^13^C‐2‐acetone molecules with different adsorption modes (217 ppm and 225 ppm) over SAPO‐34 zeolites at 298 K (Figures 1a and S5a), which can be assigned to ^13^C‐2‐acetone adsorbed on the BAS (BAS adsorption state, Type I) and the framework Al atoms (induced FLP adsorption state, Type II), respectively.^[16]^ The strong off‐diagonal correlation peaks ((217, 225) ppm, (225, 217) ppm) between those two signals in the 2D ^13^C‐^13^C proton‐driven spin diffusion (PDSD) MAS SSNMR spectrum (Figure 1b) indicates that these two types of adsorbed ^13^C‐2‐acetone are within close spatial proximity in the cage of SAPO‐34 zeolites.

Further structural information on the ^13^C‐2‐acetone adsorption modes is obtained by ^13^C‐^27^Al symmetry‐based rotational‐echo saturation‐pulse double‐resonance (SRESPDOR) experiments. As shown in Figure 1c, a more intense dipolar dephasing of 225 ppm than that of 217 ppm is observed due to the closer proximity of ^13^C‐2‐acetone and the Al atoms in the framework with Type II mode. In addition, the local coordination environment of ^27^Al is affected by the adsorption of ^13^C‐2‐acetone, which is directly reflected by the quantitative comparison of 1D ^27^Al MAS SSNMR spectrum of SAPO‐34 before and after ^13^C‐2‐acetone adsorption. For the pristine sample, a single and dominant sharp peak at 37 ppm in 1D ^27^Al MAS SSNMR spectra (black line in Figures 1d and S3a) is observed, which is assigned to the framework tetrahedral Al (Al_IV_) atoms. The slightly asymmetric peak feature is ascribed to the trace distorted framework aluminum species.^[9e]^ Upon adsorption of ^13^C‐2‐acetone, the peak becomes more asymmetric with a decreased intensity (red line in Figures 1d and S3a). Besides, an additional broad signal in the high magnetic field exists, with the corresponding changes further confirmed by 2D ^27^Al multi‐quantum (MQ) MAS SSNMR results (Figure S3b for pristine sample, Figure 1e for ^13^C‐2‐acetone adsorbed sample) According to the previous interpretation,^[22]^ the decreasing ^27^Al MAS SSNMR chemical shift is proportional to the increase of the coordination number. Therefore, the observed broad signal at a high field (30–10 ppm) is due to the adsorption of ^13^C‐2‐acetone, which shows a structural distortion of framework Al. Besides, with the increase of temperature from 298 K to 373 K (see Figures 1d and S3a), the intensity of the additional broad peak (30–10 ppm) increases gradually (broadening pink area in Figure 1d) with the expense of the main peak at 37 ppm (turning from black to red in Figure 1d), suggesting there is a transformation from the tetrahedral framework Al to the distorted one. Similar results are also observed in the ^29^Si MAS SSNMR spectra of SAPO‐34 before and after adsorption of acetone (Figure S4a). For the dehydrated SAPO‐34, peaks at −92 ppm, −96 ppm and −102 ppm can be assigned to the framework Si atoms of Si(4Al) sites, Si(3Al) sites and Si(2Al) sites, respectively.^[23]^ Upon adsorption of acetone, the peak of Si(4Al) sites at −92 ppm decreases significantly, whereas the peaks of Si species at Si(3Al) sites (−96 ppm) and Si(2Al) sites (−102 ppm) remained, demonstrating the broken of Al−O bond in the Si(4Al) sites by acetone adsorption. As a result, the proposed adsorption modes are plotted in Figure 1f, whereby Al_IV_ and Al_V_ species are attributed to Al in Type I and Type II modes, respectively.

**Figure 2:**
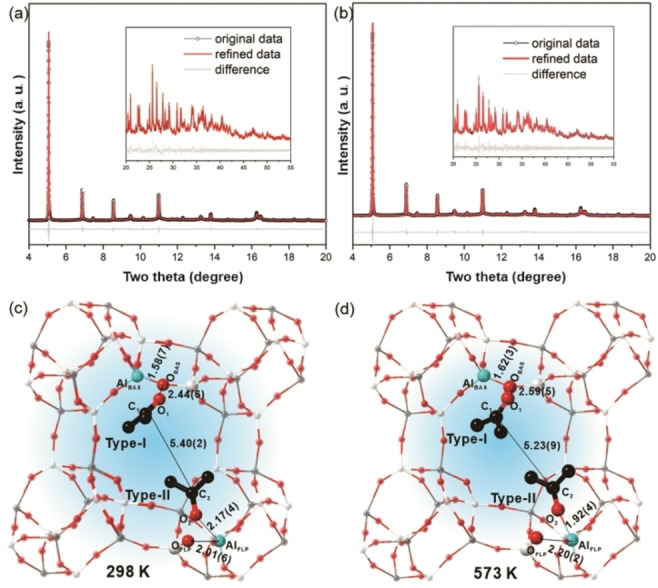
In situ VT‐SXRD patterns of acetone adsorbed on SAPO‐34 at various temperatures, the synchrotron beamline wavelength is 0.826578 Å, and the zero point is 0.000590°, Rietveld refinement profiles of SAPO‐34 pre‐adsorbed with acetone at a) 298 K and b) 573 K, with corresponding refined unit crystal models at (c) and (d) are hereby given. Their derived atomic parameters of the SXRD data by the Rietveld refinement method are summarized in Table S1. Ball‐and‐stick representation: blue=Al, red=O, white/gray=Si/P, black=C. No hydrogen atoms are plotted for clarity. Symmetry in adsorption sites is disregarded for clarity. The atomic and crystallographic parameters are summarized in Tables 1, S1, S2, and S8. In situ VT‐SXRD patterns of acetone adsorbed on SAPO‐34 at other temperatures including 323 K, 373 K, 423 K, 473 K, and 523 K, as well as their refined information can be found in the Supporting Information.

In addition to SSNMR, SXRD is a powerful technique for the high‐resolution determination of the crystallographic positions of adsorbates, such as ammonia, methanol, carbon dioxide, and ethane, in porous but long‐range ordered crystalline materials including zeolites and metal‐organic frameworks.^[24]^ The bond lengths and angles of adsorption configurations can also be derived with a high degree of accuracy. In situ experiments of acetone adsorption on SAPO‐34 at a range of temperatures using VT‐SXRD facilities were carried out at Beamline I11, Diamond Light Source, UK. The detailed information on in situ VT‐SXRD data collection can be found in the Supporting Information (Scheme S2). The temperature was increased intermittently from room temperature (298 K) to 573 K with data collected every 50 K. The data and refinement details (fitting parameters and uncertainty values) of each pattern are summarized in Figures S7–S14 and Tables S1–S8. Despite the increasing thermal motions with higher uncertainties, all the VT‐SXRD data can still be refined well with low R‐factors of excellent data‐fitting values (Table S1). By comparing VT‐SXRD patterns of the acetone before and after adsorption over SAPO‐34, it is found that the relative intensities of the peaks are changed upon the addition of acetone, but without inducing new peaks or any significant peak broadening (Figure S6). This suggests that the acetone adsorption does not induce a major alteration to the parent SAPO‐34 structure. In all the Rietveld‐derived structures (Figures 2 and S7–S13), the same positions of acetone sites can be repeatedly yielded by convergence during refinement from various distances or angles relative to the framework upon numerous attempts.

**Figure 3:**
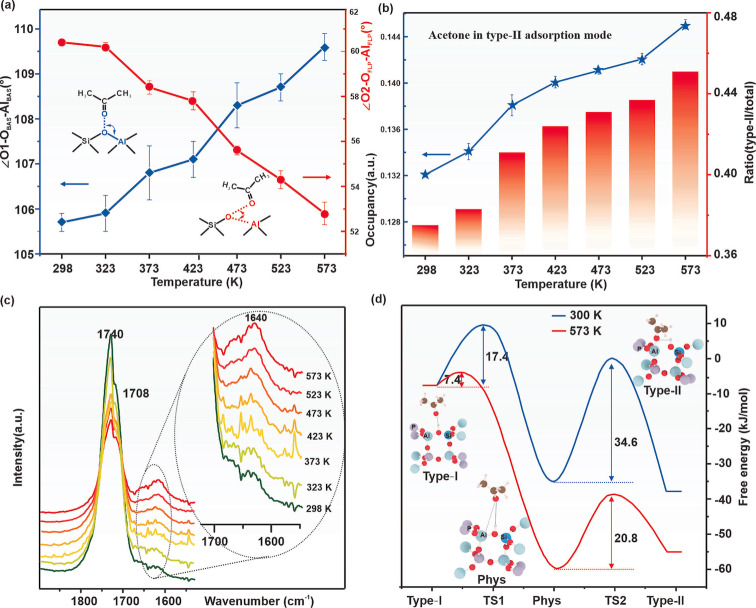
a) The adsorption angle (∠O_1_‐O_BAS_‐Al_BAS_, Type I mode in red, ∠O_2_‐O_FLP_‐Al_FLP,_ Type II mode in blue) changes, b) the SOF of acetone in Type II mode, and the ratio (Type II/total) as a function of temperature in the range of 298 K to 573 K based on Table 1 by Reitveld refinement of VT‐SXRD, c) in situ DRIFT spectra of acetone adsorbed on SAPO‐34 heated from 298 K to 573 K, an inset displays the enlarged region of 1700–1600 cm^−1^. The data were collected on a Nicolet iS50 FT‐IR spectrometer with a high‐temperature, high‐pressure DRIFT reaction cell using an MCT/A detector, 64 scans per data point, and at a resolution of 4 cm^−1^, d) potential energy surfaces of the transformation from BAS to induced FLP by acetone adsorption at 300 K and 573 K, details of the calculations are available in Supporting Information.

Figures 2a and 2b display the calculated patterns yielded by Rietveld refinement of acetone adsorbed on SAPO‐34 at 298 K and 573 K, and the corresponding structure solutions are shown in Figures 2c and 2d, respectively. Indeed, we could clearly see the two types of adsorption modes for acetone over SAPO‐34 based on SXRD refinements at 298 K and 573 K (Figures S7 and S13 for details). This is in agreement with the SSNMR data in Figure 1, except that in the refined SXRD structure the Type II acetone is leaning closer to Al (induced FLP) at 573 K than that at 298 K. As a result, the progressive structural changes in refined parameters such as bond length, angles and site occupancy factor (SOF, the measure of statistical chance of the site occupation) are particularly analyzed at the increased temperatures. As shown in Table 1, we have found that the adsorption modes both in Type I and Type II modes remain almost unchanged below 323 K, as indicated by the very small change in bond lengths, angles, and SOF. Whereas upon increasing the temperature above 373 K, the bond lengths of Al_BAS_‐O_BAS_, O1‐O_BAS_ in Type I mode, and Al_FLP_‐O_FLP_ in Type II mode show an appreciable lengthening, accompanied by the shortening of O2‐Al_FLP_ in Type II mode. Additionally, the acetone in the Type I mode shows a gradual departure from the BAS to the center of the pore as shown by the shortening C1–C2 distance (ca. 5.39(3) Å at 323 K to ca. 5.23(9) Å at 573 K). The observation of Type I mode can be consistent with the expected lengthening of the interatomic distances arising from thermal expansion at higher temperatures, but Type II mode is in stark contrast to the Type I mode. Instead of the lengthening of interatomic distance in Type I mode, the acetone in Type II mode is found even closer to the framework aluminum as the distance of O2⋅⋅⋅Al_FLP_ becomes significantly shorter (ca. 1.92(4) Å at 573 K vs ca. 2.09(2) Å) at 373 K). This unique observation of Type II mode clearly depicts a strengthening of the adsorbate‐framework interaction inside the cage of SAPO‐34. Moreover, the distance of Al_FLP_‐O_FLP_ is increased significantly at high temperatures (2.20(2) Å at 573 K), which indicates that there is a degree of distortion/reconstruction of surface framework Al_FLP_ after acetone adsorption. Apart from the variation of bond length, the progressive changes in angles of Type I and Type II modes are also noted (Table 1). Figure 3a shows the adsorption angle changes (∠O_1_‐ O_BAS_‐ Al_BAS_ marked in blue for Type I mode, ∠O_2_‐O_FLP_‐Al_FLP_ marked in red for Type II mode) as a function of temperature in the range of 298–573 K. In Type I mode, ∠O_1_‐O_BAS_‐ Al_BAS_ exhibits a clear trend of increase angle from 298 K to 573 K due to thermal motion. However, a decreasing trend in ∠O_2_‐O_FLP_‐Al_FLP_ is observed in Type II mode with increasing temperature, indicating the possible temperature‐promoted conversion of chemisorbed acetone from Type I to Type II state of increasing population. As for SOF, despite the increasing thermal factors (greater errors in measurements as seen in increasing Beq values, see Tables S2–S8), a notable progressive decrease in the SOF of acetone in the Type I mode is observed, due to desorption and conversion to Type II mode from 298 K to 573 K (Figure S6c). In contrast, the SOF of acetone in Type II mode increases rather irregularly from contrasting effects of Type I mode conversion and desorption (Table 1). The dynamic change of the SOF and the proportion of adsorbed molecules in Type II mode/total mode is plotted in Figure 3b as a function of temperature. A gradual accumulation of the acetone in Type II mode (blue line in Figure 3b) but a decrease of acetone in Type I mode (orange line in F Figure S6c) is observed, suggesting that at high temperature, acetone adsorption in Type II mode is preferable. Thus, it is apparently conceivable that the Type II mode of acetone is formed at the expense of the Type I mode of acetone at increased temperatures (Figure 3b and Figure S6c), although the decrease in SOF due to the desorption at elevated temperature is ineluctable.^[25]^

**Table 1:**
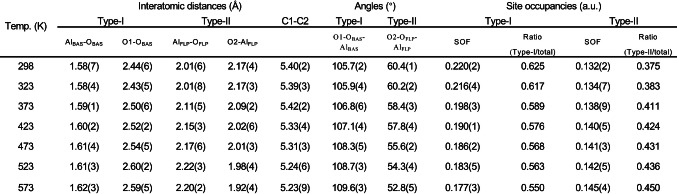
Derived interatomic distances, angles and site occupancies from Rietveld refinements of two types of acetone adsorption modes in the cage of SAPO‐34. The numbers in the brackets are the estimated standard deviations.

FT‐IR is an effective analytical tool to characterize atom vibration in different chemical environments.^[26]^ Thus the two different acetone adsorption modes and their transformation at different temperatures were also examined by in situ DRIFT in this work (Figure 3c under atmospheric pressure condition and Figure S14a under a dynamic vacuum to reduce the signals of gas phase/physisorbed acetone). Experimental details are available in the Supporting Information. As shown in Figure 3c, sharp and strong bands can be found at ca. 1740 cm^−1^ and ca. 1708 cm^−1^, as well as a considerably weaker band at ca. 1640 cm^−1^. According to literature,^[27]^ the dominant peak at ca. 1740 cm^−1^ is characteristic of *v*(C=O) in gaseous acetone,^[26c]^ while the absorption peak at ca. 1708 cm^−1^ originates from the C=O stretching vibration modulated by H^+^ via the interaction of acetone with the BAS (acetone in Type I mode).^[26c]^ The band at ca. 1640 cm^−1^ are assigned to acetone that interacts with the Al Lewis acid sites,^[26a]^ thus attributed to acetone adsorbed on the induced FLP sites (acetone in Type II mode). With the increase of temperature from 298 K to 573 K, the peak at ca.1708 cm^−1^ exhibits a decrease in intensity due to the acetone desorption and conversion to another form(s), but the peak at 1640 cm^−1^ shows a pronounced and continuous increase presumably from the Type I mode, as shown in the inset of Figure 3c (cf. desorption study at 573 K in Figure S14b–c). The results are in good agreement with both the solid‐state ^27^Al MAS SSNMR results (Figure 1d) and the in situ VT‐SXRD data (Figure 3b) in that there is a temperature‐induced transformation of acetone from Type I to Type II adsorption modes. All of the above results suggest that FLP adsorption of acetone is more favorable at increased temperatures. Thus, for acetone of given polarity, a strong thermal effect can promote the transformation from the BAS mode to the FLP adsorption mode. It should also be noted that because the experimental conditions of SSNMR, VT‐SXRD, and in situ DRIFT were not exactly identical, we could not directly use the quantitative results from different techniques to cross‐verify the Type I and Type II proportion under different temperatures, but the trend that the promoting transformation of acetone in Type II mode from Type I mode with increasing of temperature is convincible based on these experimental techniques.

To further investigate the changes in adsorption structures of acetone on SAPO‐34 under different temperatures, advanced ab initio molecular dynamic (AIMD) simulations were also conducted (details of calculation methods and parameters are provided in the Supporting Information). Our modeling results derived from AIMD simulations of acetone in SAPO‐34 zeolite at different temperatures indeed support the above experimental observations. Figure 3d shows the structures of two adsorption modes and their free energy surfaces projected from minimum free‐energy paths at 300 K and 573 K, in which two steps are involved for the transformation of acetone from Type I to Type II mode. The results suggest that acetone in Type II mode is more stable than in Type I mode, and the transformation from Type I to Type II mode becomes even more favorable at higher temperatures by lowering the free energy barriers. In the simulation at low temperature (Figure 3d, 300 K in blue), an energy barrier of 34.6 kJ mol^−1^ for the transformation from Type I to Type II mode is calculated. However, the increase in temperature could greatly promote the transformation with a lower energy barrier of 20.8 kJ mol^−1^, presumably due to the induced structural reconfiguration (Figure 3d, 573 K in red). The detailed dynamic transformation pathways between acetone in Type I and Type II mode over SAPO‐34 under 300 K and 573 K as derived by AIMD simulation are displayed in Movies S1and S2, respectively. Although acetone is frequently partitioned between the Type I, physical adsorption, and Type II boundary states, a larger population of acetone in the Type II state is more obvious, as reflected by the free‐energy surface profile (Figure 3d). The changes in bond angles (∠O_1_‐O_BAS_‐Al_BAS_, ∠O_2_‐Al_FLP_‐O_FLP_) and lengths (O_1_‐O_BAS_, O_2_‐Al_FLP_) as a function of evolution time are plotted in Figure S15. Despite the fluctuation in bond angles and lengths, at ≈15 ps, there are sharp changes in the average angles of ∠O_1_‐O_BAS_‐Al_BAS_ and ∠O_2_‐Al_FLP_‐O_FLP_ in Figures S15a, which exhibit the opposite trends at 300 K: ∠O_1_‐O_BAS_‐Al_BAS_ becomes smaller when acetone moves from BAS adsorption mode to induced FLP mode, whereas ∠O_2_‐Al_FLP_‐O_FLP_ increases due to surface reconstruction accordingly. For the bond lengths, shown in Figure S15c, there is also an expected decrease in O_2_‐Al_FLP_, but the data are much more blurred by the thermal motions. At 573 K (Figures S15b and S15d), it takes a shorter time (≈10 ps) for the changes to take place, indicating the higher rate for the conversion amid the apparent desorption of the acetone species. Notice that these temporal and spatial changes in angles and bond lengths of acetone on dynamic conversion from BAS (Type I mode) to FLP (Type II mode) at fixed temperature by AMID are different from the study of equilibrium positions of acetone in Type I and Type II mode by the VT‐SXRD in Figure 3a. Thus, the theoretical calculations provide a molecular basis for the structural reconfiguration in adsorbate‐adsorbent in agreement with our experimental observations, showing that more induced FLP adsorption modes are created from BAS modes with increasing temperature.

## Conclusion

In summary, using acetone as a probe molecule, two types of adsorption modes, namely the BAS adsorption mode and induced FLP adsorption mode are confirmed under various temperatures. SSNMR, in situ VT‐SXRD, and in situ DRIFT experiments reveal that increasing temperature can promote the transformation of BAS to induced FLP adsorption sites, which is consistent with theoretical calculations based on AIMD simulations. Our work sheds light on the adsorbate and temperature‐induced dynamic active site modification in zeolites, which we hope will stimulate further works in this area.

## Conflict of interest

The authors declare no conflict of interest.

1

## Supporting information

As a service to our authors and readers, this journal provides supporting information supplied by the authors. Such materials are peer reviewed and may be re‐organized for online delivery, but are not copy‐edited or typeset. Technical support issues arising from supporting information (other than missing files) should be addressed to the authors.

Supporting Information

Supporting Information

Supporting Information

## Data Availability

The data that support the findings of this study are available in the Supporting Information of this article.
