# Supplementary material for: Thermal Alteration in Adsorption Sites over SAPO‐34 Zeolite
Source: Angew Chem Int Ed Engl. 2022 May 12;61(27):e202204500. doi: 10.1002/anie.202204500 (PMC9322573; doi:10.1002/anie.202204500)
Supplement: Supplementary file 1 — Supporting Information [file ANIE-61-0-s001.pdf]

Angewandte Chemie
Eine Zeitschrift der Gesellschaft Deutscher Chemiker

Supporting Information

Thermal Alteration in Adsorption Sites over SAPO-34 Zeolite

G. Li, T. Yoskamtorn, W. Chen, C. Foo, J. Zheng, C. Tang, S. Day, A. Zheng, M. M.-J. Li, S. C. E. Tsang**

This file includes:

Experimental details, methods, additional results of SSNMR spectra, in-situ SXRD structure refinement data, in-situ DRIFT results and AIMD simulations including:
Figures S1- S15
Schemes S1- S3
Tables S1- S8
Movies S1-S2

1. Materials and Methods

1.1 Materials

Zeolites of SAPO-34 were obtained from ACS Materials, USA. Chemicals including acetone and ^{13}C-2-acetone were commercially available (Sigma-Aldrich) with 99 % purity or above, and used as received.

1.2 Solid-state NMR experiments

SAPO-34 was performed on a vacuum line (Scheme S1a) for dehydration before adsorption of ^{13}C-2-acetone. The temperature was gradually increased at a rate of 1K/min and the samples were kept at a final temperature of 673 K at a pressure below 10^{-3} Pa over 10 h. After the samples cooling to ambient temperature, a certain amount of ^{13}C-2-acetone was introduced into the activated samples. Finally, the sample tubes were flame sealed. Prior to NMR experiments, the sealed sample was transferred into a ZrO_2 rotor with a vespel endcap under a dry nitrogen atmosphere in a glovebox.

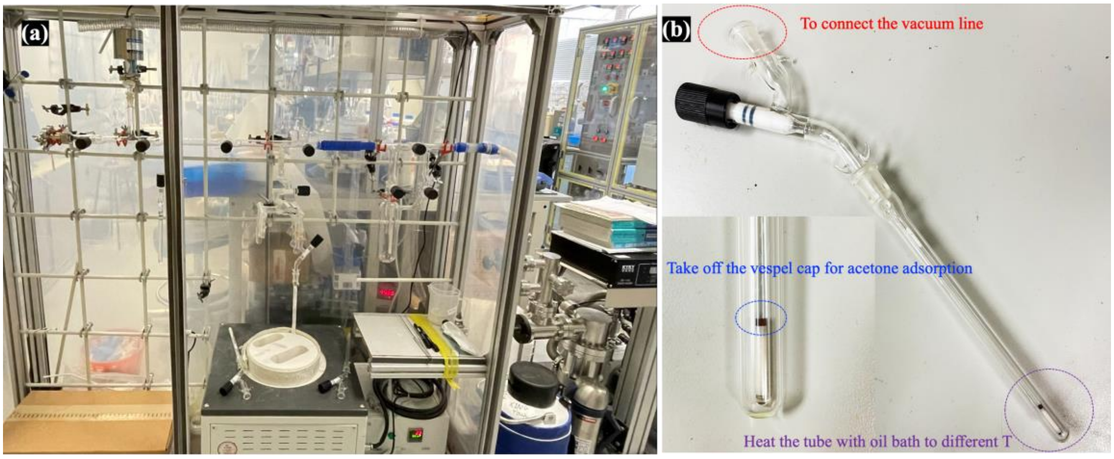

Scheme S1. (a) The device of high vacuum system for samples' dehydration and probe adsorption, (b) sealed glass tube device for sample dehydration, acetone adsorption and rotor heating.

Once the NMR scanning of the dehydrated sample was completed, the sealed rotor was then transferred to a glove box. In the glove box, vespel cap was removed. And the

rotor (with sample) was then placed into a glass tube (Scheme S1b). Sealed the glass tube before taking it out of the glove box. The glass tube was connected to the high vacuum line for pumping out the residual N_2 in the glass tube, followed by introducing acetone for adsorption. After that, the glass tube was sealed and transferred into the glove box. The rotor was taken out from the glass tube and sealed with the same vespel cap in the glove box before sending for the NMR measurement under identical scan parameters. After finishing the room temperature scanning, the NMR rotor with acetone adsorbed sample was transferred into a glass tube (Scheme S1b). Then the glass tube was heated to 323 K in an oil bath and held for 30 mins before cooling down to the room temperature. After that, the sealed rotor was loaded into the NMR probe again for scans under the same parameters. Repeat the same procedures by heating the sealed rotor to 348 and 373 K for collecting the results at various temperatures.

^{27}Al, ^{29}Si, ^{31}P MAS NMR experiments were carried out on a JEOL ECZ-500R spectrometer with the Larmor frequencies of 130.28, 99.34, 202.4 MHz, respectively. ^{27}Al one pulse MAS NMR spectrum were recorded with a pulse length of 0.12 μs (<π/12), a recycle delay of 1 s, and a spinning rate of 12 kHz using 3.2 mm rotor. ^{29}Si MAS NMR spectra were recorded on 8 mm rotor with a spinning rate of 4 kHz, a π/2 pulse length of 5.2 μs, and a recycle delay of 60 s. ^{29}Si MAS NMR of SAPO-34 before and after adsorption of acetone were both carried out. ^{31}P one pulse MAS NMR spectra were recorded on 3.2 mm rotor with a pulse length of 2.75 μs (π/2), a recycle delay of 30 s, and a spinning rate of 12 kHz.

^{27}Al -^{27}Al multiquantum (MQ) MAS NMR

^{27}Al MQ MAS spectra was performed on JEOL ECZ-500R spectrometer with the pulse sequence proposed by Amoureux et al. (1) 312 scans were accumulated and the recycling delay was set to 1.0 s. Hyper-complex method was used in the 2D ^{27}Al MQ MAS data acquisition and processing. The MAS spin rate was 12 kHz in ^{27}Al single-pulse and MQ MAS experiments using 3.2 mm rotor.

1D ^{1}H, ^{13}C cross-polarization (CP) and all the 2D MAS NMR experiments were performed on Bruker Avance III 400 WB spectrometer at resonance frequencies of 399.33 MHz and 104.22 MHz for ^{1}H and ^{13}C, respectively. ^{1}H MAS NMR spectra were acquired on a 4 mm rotor with a spinning rate of 10 kHz, a π/2 pulse length of 3.8 μs, and a recycle delay of 5 s, while the samples used for the ^{1}H MAS NMR studies were additionally dehydrated in vacuum at 673 K for 10 h before the measurements or the loading with the adsorption molecules. ^{13}C CP MAS NMR were acquired on a 4 mm probe with a contact time of 4 ms, a recycle delay of 2.5 s, and a sample spinning rate of 10 kHz.

2D ^{13}C-^{13}C proton-driven spin diffusion (PDSD) MAS NMR experiments were performed to determine the correlations between ^{13}C signals. ^{1}H → ^{13}C cross polarization with a contact time of 4 ms was used to prepare the initial ^{13}C signal. The π/2 pulse width of ^{13}C is 4.9 μs and the mixing time was set to 100 ms. A TPPM-13 ^{1}H decoupling at 62.5kHz was applied to the acquisition time t2. The experiments were acquired with 64 scans per experiment and 360 experiments in the State-TPPI scheme.

^{13}C-^{27}Al symmetry-based rotational-echo saturation-pulse double-resonance (SRESPDOR) MAS NMR experiments were carried out with the help of a frequency splitter (REDOR-BOX), which enables tuning and matching to the Larmor frequencies of the ^{13}C and ^{27}Al nuclei on the X channel of 4mm HXY MAS NMR probe at the same time. The initial ^{13}C signal was prepared by $^{1}H \rightarrow {}^{13}C$ cross polarization (same condition as the previous ^{1}H-^{13}C CP MAS experiments). SR4 dipolar recoupling was used on the ^{13}C channel with $\nu_{nut,13C}$ =20 kHz. Continuous-wave ^{1}H decoupling with the amplitude of 62.5 kHz was used during SR4, while a SPINAL-64 (small-phase incremental alternation with 64 steps) ^{1}H decoupling with the amplitude of 62.5 kHz was used during acquisition. A π pulse length of 8 μs was used on the ^{13}C channel. The saturation pulse on the ^{27}Al channel was divided into two pulses with duration of 75 μs (0.75 Tr) to transfer ^{13}C-^{27}Al interactions. The recycle delay was set to 3 s.

1.3 In situ Variable Temperature Synchrotron Powder X-ray diffraction (VT-SXRD)

VT-SXRD data were collected at Beamline I11, Diamond Light Source, UK. The energy of the incident X-ray flux was set at 15 keV. The wavelength and 2θ-zero point were refined using a diffraction pattern obtained from a high-quality silicon powder (SRM640c). The beamline wavelength is 0.826578 Å, and the zero point is 0.000590° Under room temperature, the pristine zeolite powder was loaded in a 0.7 mm borosilicate glass capillary. As for in situ work, sample was loaded in a capillary gas cell. A capillary reactor cell was specifically designed for this experiment with SAPO-34 sample under operando VT-SXRD (Scheme S2). The stainless-steel capillary cell support holder was strong and rigid enough to ensure the alignment of the capillary for low and high temperature experiments. A both-way open-ended borosilicate capillary (diameter of 0.5 mm) was mounted by two brass capillary holders and was glued using araldite; over the reaction temperature and pressure (up to 10 bar); no gas leakage was detected. Within the both-way open-ended capillary, the SAPO-34 sample was sandwiched between two pieces of quartz wool in the capillary to prevent sample dislocation. One end of the capillary was connected to a gas inlet end that received anhydrous acetone vapor from a liquid saturator kept at room temperature with He carrier gas that has a flow rate of 2 mL min^{-1}. From the thermodynamics perspective, the environment in the bubbler must have reached equilibrium. The partial pressure of acetone in the gaseous section of the bubbler should equate to the partial pressure of acetone at the specific temperature, i.e. 0.167 atm at 25 °C. There is sufficient time for the environment inside the bubbler to reach dynamic equilibrium throughout the entire VT-SXRD experiment. As a result, the outlet acetone containing gas should possess a partial pressure about 0.167 atm. The vapor pressure of acetone and the flow of He carrier gas were carefully calibrated to ensure a reasonable amount of acetone vapor reaching the sample. For the heating of the sample, a hot-air blower (RT-1000°C) was mounted at 5 mm from the capillary. The temperature of the hot-air blower had been calibrated against Pt powder sample. Therefore, a constant capillary rocking generated by the sample stage holder was employed to reduce the loss in data quality.

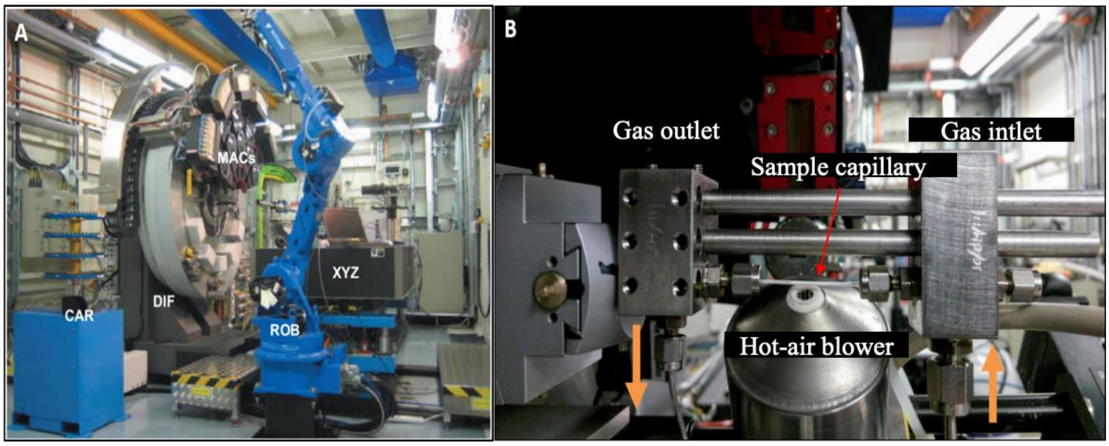

Scheme S2. Operando VT-SXRD set-up beamline I11 at Diamond Light Source, UK. (a) 5 arms (x9) multiple-analyser crystals (MAC) detectors of diffractometer (DIF) with a carousal auto-sampler and a capillary sample stage. (b) The high-pressure operando gas-cell system.

High resolution data were obtained from the samples using the multi-analyser crystal (MAC) detectors. The patterns were collected in the 2θ range 0-150 ° with 0.001° data binning. Each pattern was collected for an hour for good statistics.

The diffraction patterns were analyzed by Rietveld refinement methods based TOPAS (V6) to obtain structural details. The starting coordinates were based on the SAPO-34 model in our previous work (2). The Thompson-Cox-Hastings pseudo-Voigt peak function was applied to described the diffraction peaks. The scale factor and lattice parameters were allowed to refine for all the diffraction patterns. The refined structural parameters for pattern were the fractional coordinates (x, y, z) and isotropic displacement factors (Beq) for all atoms, and the site occupancy factors (SOF), translation and rotation axes for the rigid body Z-matrices describing the adsorbed ammonia molecules within the SAPO-34 framework. The quality of the Rietveld refinements of synchrotron data has been assured with a low goodness-of-fit (Gof) factor, a low weighted profile factor (Rwp) and a well fitted pattern with acceptable temperature factor (Beq) within experimental errors. During the refinement, all the T-sites (T=Al, Si, P) of SAPO-34 were set to share the same value of SOF and Beq. All the O-sites of SAPO-34 were set to share the same value SOF and Beq. The crystallographic data and refinement details are summarized in Table S1**.**

1.4 In situ diffuse reflectance infrared Fourier transform spectroscopy (DRIFTS) experiments

In situ DRIFTS experiments were performed in the range of 650-4000 cm^{-1} on a Nicolet iS50 FT-IR spectrometer with a high temperature, high pressure DRIFTS reaction cell using an MCT/A detector at a resolution of 4 cm^{-1}(Scheme S3a-b).Approximately 50 mg of H-SAPO-34 zeolite (after calcination in air at 823 K for 6 h) was placed in the DRIFTS cell and activated under a dry N_2 flow (10 mL/min) at 673 K (with linear heating to 673 K for 2 h and holding for 24 h) to remove any adsorbed water molecules. The sample cell was then cooled down to room temperature. During the cooling process, background information of the pristine H-SAPO-34 were collected at different temperatures (50 K a point). At room temperature, a constant flow of N_2 (10 mL/min) was passed through a flask of acetone solution and subsequently bubbled into the sample cell for 20 mins to achieve saturated adsorption of acetone inside the zeolite sample. After that, the dry N2 with the same flow rate was re-introdeced into the system for 120 mins to purge extra-adsorbed acetone, Similar procedure was applied for the data collection during the cooling period. The timeline of the data collection is shown in Scheme S3c. Each spectrum was an average of 64 scans.

We have also conducted the DRIFT experiment under a dynamic vacuum condition, in which most of procedures are similar to the above, except that a pump with a valve was connected to the in situ cell after the acetone adsorption in order to reduce the signals from gas phase or physisorbed acetone as quick as possible after the flowing of acetone. Then, the sample cell was gently heated to different target temperatures to record the data

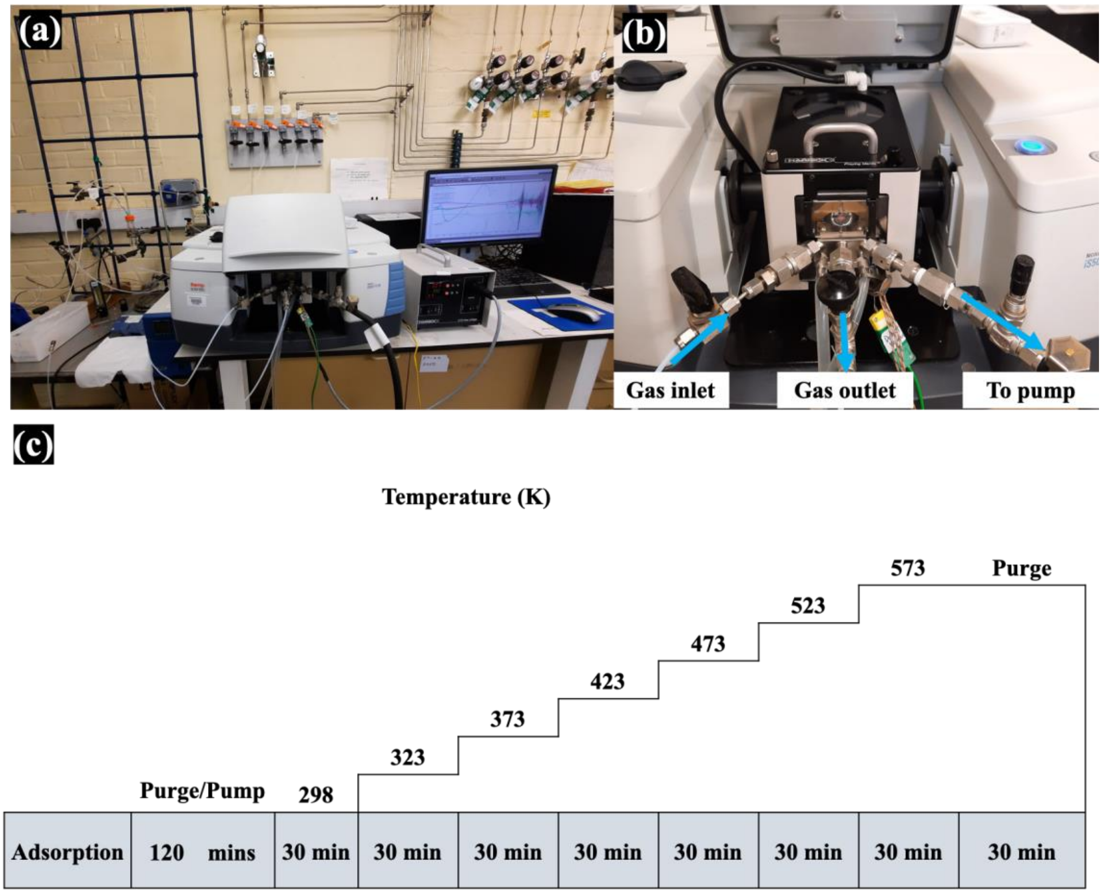

Scheme S3. (a) The device of in-situ DRIFT spectrometer, (b) in-situ cell, (c)timeline of the data collection for the in-situ DRIFTS experiments.

1.5 Ab inito molecular dynamic (AIMD) simulations

AIMD simulations were performed by periodic density functional theory (DFT). Firstly, equilibrium AIMD simulation of reactant state species were carried out 5 ps at NPT ensemble to relax the cell parameters and atoms positions. Next, the metadynamics (MTD) methods were performed to explore the acetone adsorption in H-SAPO-34 in AIMD simulations.

All the AIMD simulations were employed by CP2K software, and the linked PLUMED code was used to carry out the MTD simulations. The PBE functional (3) with consideration of Grimme's D3 dispersion corrections (4) was chosen for the DFT calculations. The double-ζ valence plus polarization DZVP basis set (5) and Goedecker-Teter-Hutter (GTH) pseudopotential (6) was used for the system. During the SCF procedure, a 360 Ry density CUTOFF criterion with the finest grid level was employed, together with multi-grids number 4 (NGRID 4 and REL CUTOFF 70). The temperature of AIMD was controlled by a chain of five Nosé-Hoover thermostats, (7) and the integration time step was set to 0.5 fs.

Metadynamics (MTD) simulation (8) was employed to explore the methanol dehydration over Lewis acidic sites. This is done by first optimizing the geometrical structures and cell parameters of the configuration by periodic DFT followed by a 5 ps NPT molecular dynamics simulation to relax the structure and cell parameters under experimental conditions such as temperature and pressure. Accordingly, MTD simulations were performed at 300 K in NVT ensemble, and this advanced sampling technique is used to enhance the probability of sampling chemical reactions or rare events provided that a limited number of collective variables (CV) describing the reaction coordinate were well-defined. Nonetheless, MTD simulation is normally biased by regularly spawning Gaussian hills along the chosen CVs, which may be defined by coordination numbers (CN):

$$\mathrm{CN} = \sum_{ij} \frac{1 - \left(\frac{r_{ij} - d_0}{r_0}\right)^n}{1 - \left(\frac{r_{ij} - d_0}{r_0}\right)^m}$$

where r_{ij} represents the interatomic distance between atoms i and j and r0 is the reference distance. In this work, parameters d0, n and m were set to 0, 6, and 12, respectively.

2 Supplementary results

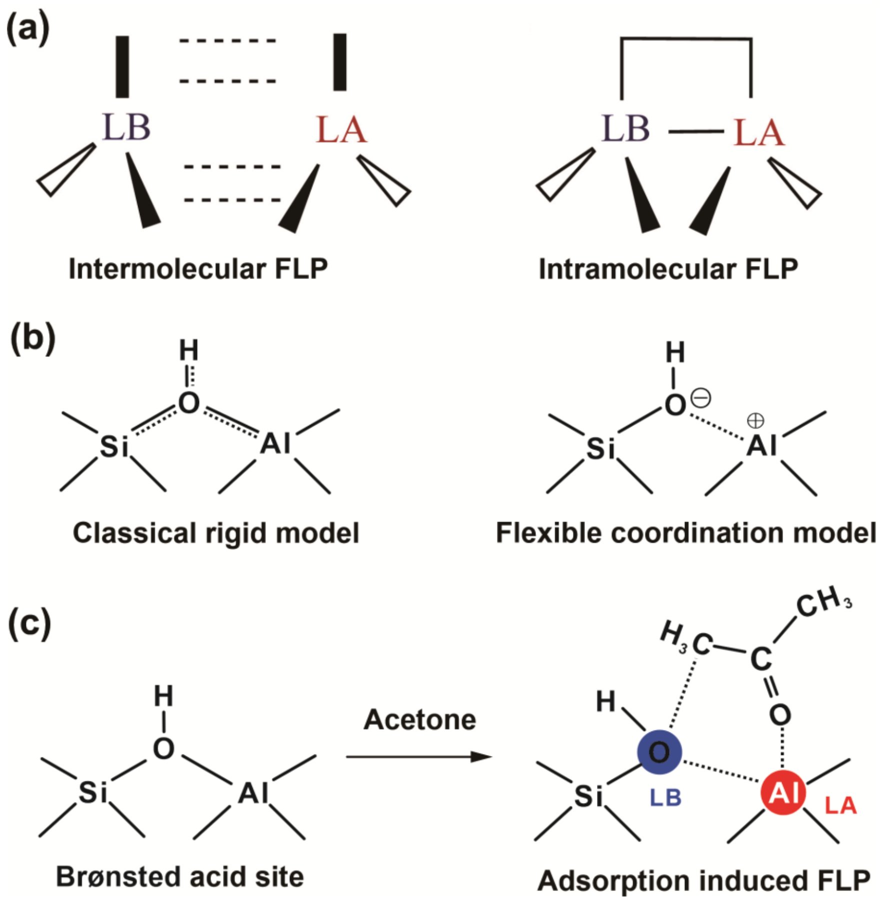

Figure S1. (a) Schematic image of traditional Frustrated Lewis pair (I: intermolecular FLP, II: intramolecular FLP, LA for Lewis acid, LB for Lewis base) (9), (b)Brønsted acid sites in zeolites represented as a resonance hybrid between two structures (10), (c)acetone adsorption induced Frustrated Lewis pair in SAPO-34 zeolite in this work.

In general, there are two types of FLP (Figure S1a), one is intermolecular FLP (the LA and LB centers are contained in two separate molecules), the other one is intramolecular FLP (the reactive centers in intramolecular FLPs are covalently connected to each other, with LA–LB distances that resemble that of the classic Lewis adducts). In zeolites, Bronsted acid sites are regarded as a hybrid structure (Figure S1b), including (i) a fully bridged oxygen with a weakly bound proton (classical rigid model), and (ii) a silanol group interacted with a weak Lewis acid Al through the hydroxyl oxygen (flexible coordination model). In this work, a newly discovered FLP adsorption geometry will be induced upon the adsorption of polar molecules (Figure S1c). Taking acetone adsorbate as an example: the Lewis basic atom of the acetone is directly bonded to the Lewis acidic Al site, while the Lewis acid atom of the acetone is directly bonded to the Lewis basic SiO(H).

2.1 SSNMR results

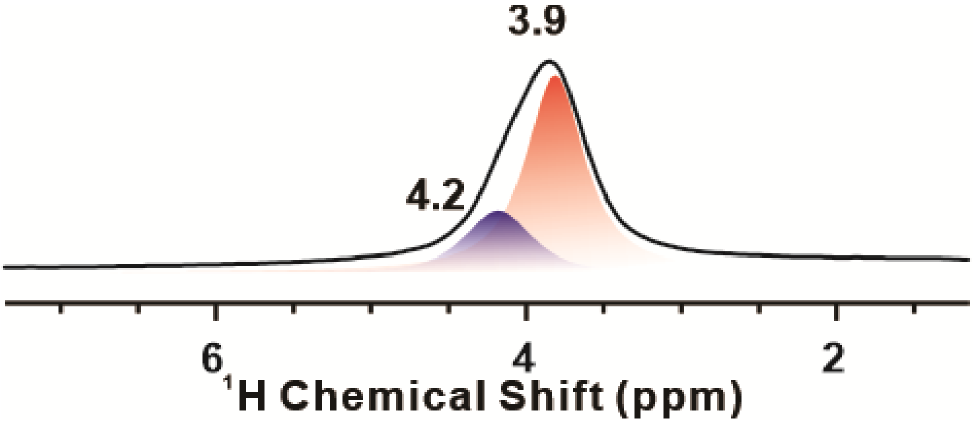

Figure S2. ^{1}H MAS SSNMR of dehydrated pristine SAPO-34.

^{1}H MAS chemical shifts of SAPO-34 is shown in Figure S2, two peaks at 4.2 ppm and 3.9 ppm are observed. According to the previous literature (11), both of them can be unambiguously assigned to the bridging hydroxyl.

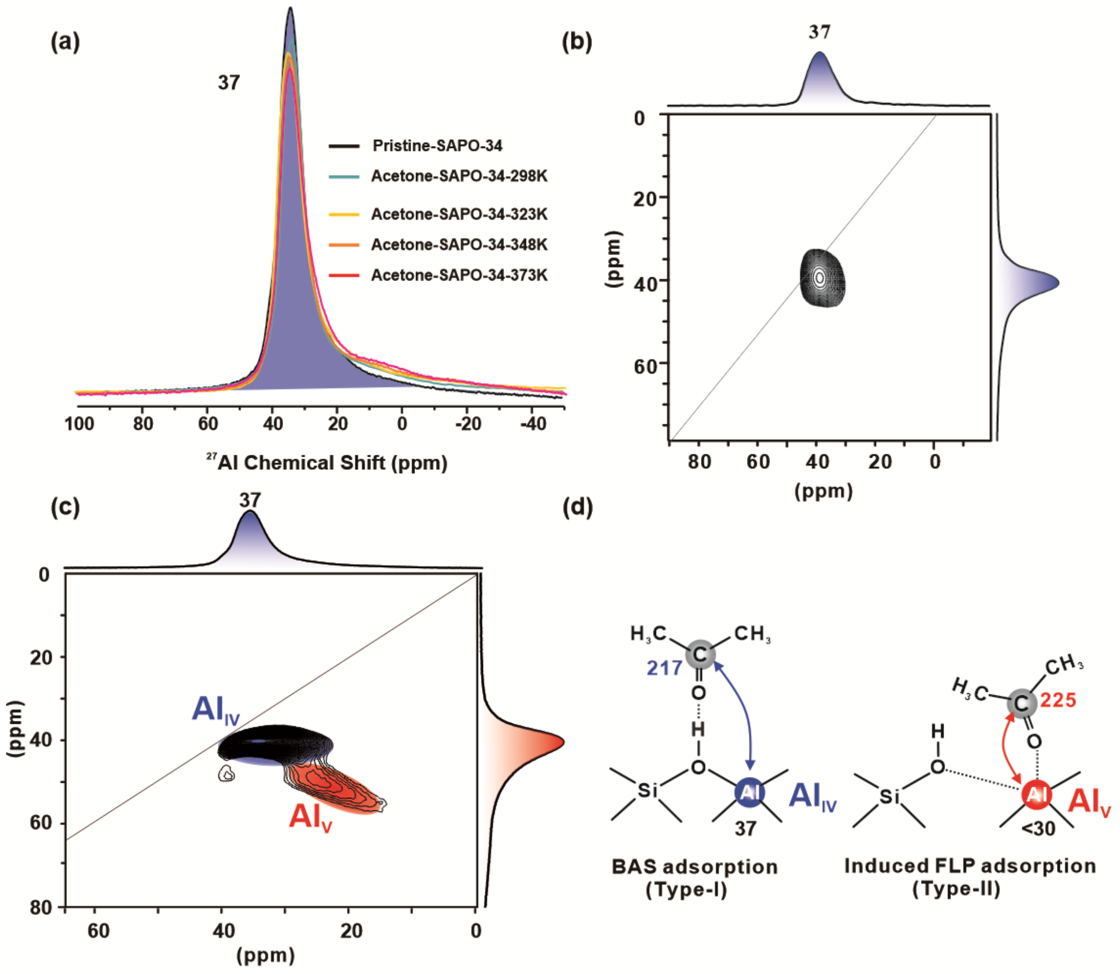

Figure S3. (a) 1D ^{27}Al MAS SSNMR spectra of SAPO-34 before and after adsorbed with ^{13}C-2-acetone under 298K, 323K,348K and 373K, (b) 2D ^{27}Al MQ MAS SSNMR of dehydrated pristine SAPO-34, (c) ^{27}Al MQ MAS SSNMR spectrum of SAPO-34 adsorbed with ^{13}C-2-acetone at 298 K, (s) proposed acetone adsorption modes in SAPO-34.

Figure S3a shows the changes of ^{27}Al chemical shifts of ^{13}C-2-acetone before and after adsorbed on SAPO-34 zeolites under different temperatures from 298 K to 373 K. For pristine sample, single and symmetrical peak at 37 ppm can be attributed to the framework tetrahedral aluminum species (Al_{IV}). Upon the adsorption of ^{13}C-2-acetone, an additional broad peak at 30-10 ppm occurs, and the corresponding peak intensity is enhanced gradually with the increasing temperature. According to the previous literature (12), the decreasing ^{27}Al NMR chemical shift is proportional to increasing the coordination numbers, therefore, the observed broad signal in the high field (30-10 ppm), due to the adsorption of ^{13}C-2-acetone, would mean that there is a structural distortion of framework Al. In other words, the framework Al local chemical environment has been altered by the absorption of ^{13}C-2-acetone. Noted that the same sample was used before and after the acetone adsorption (same pristine sample was exposed to ^{13}C-2-acetone), it is clear that adsorption of ^{13}C-2-acetone can strongly affect the coordination of framework Al. This adsorbate induced structure change is

confirmed by the 2D MQ ^{27}Al MAS SSNMR experiments (Figures S3b and S3c) that an additional correlation peak arises after the ^{13}C-2-acetone adsorption. The peak of Al_{IV} resonates close to the diagonal, indicating that the corresponding Al species experience a small quadrupolar interaction. On the other hand, the peak Al_{V} (Figure S3c red) deviates from the diagonal, indicating a large anisotropic quadrupolar-induced shift and broadening. Thus, we assign the broad peak (Figure S3c red) to the framework Al_{V} with the adsorption of ^{13}C-2-acetone. As a result, the proposed adsorption modes are illustrated in Figure S3d, whereby Al_{IV} and Al_{V} represent Al in Type-I and Type-II modes, respectively.

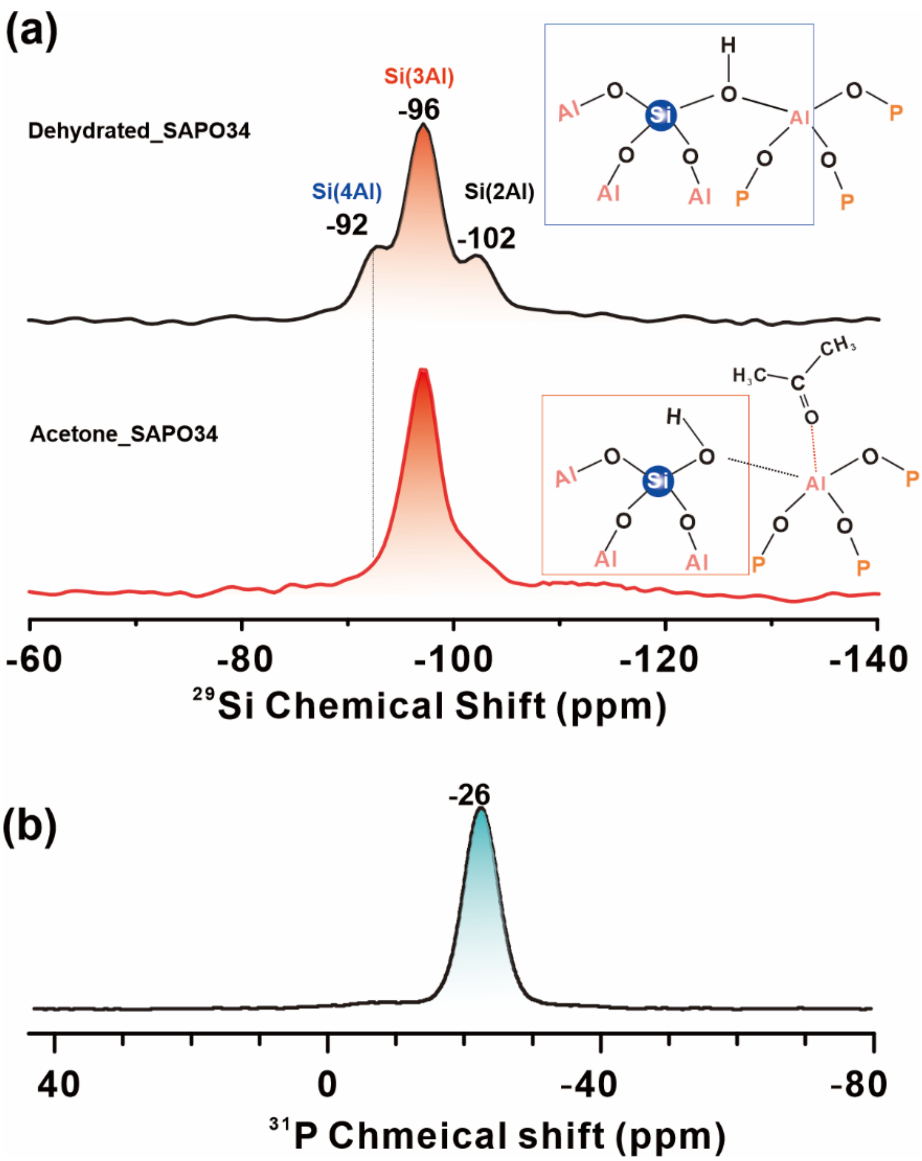

Figure S4. (a) ^{29}Si MAS SSNMR of SAPO-34 before(black) and after(red) adsorption of acetone, (b) ^{31}P MAS SSNMR of hydrated pristine SAPO-34.

^{29}Si MAS NMR spectra of SAPO-34 before(black) and after(red) adsorption of acetone are displayed in Figure S4a. According to the literature, peaks at -92 ppm, -96 ppm and -102 ppm can be assigned to the framework Si atoms of Si(4Al) sites, Si(3Al) sites and Si(2Al) sites, respectively(13). Upon adsorption of acetone, peak of Si(4Al) sites(-92 ppm) decreases significantly, while the peaks of Si species at Si(3Al) sites and Si(2Al) site remained, suggesting the change of local environment of Si(4Al) sites by acetone adsorption, as reflected in the right hand side in Figure S4a. Figure S4b displays the ^{31}P MAS SSNMR spectrum, single and sharp peak at -26 ppm can be attributed to the tetrahedrally coordinated framework phosphorus atoms.

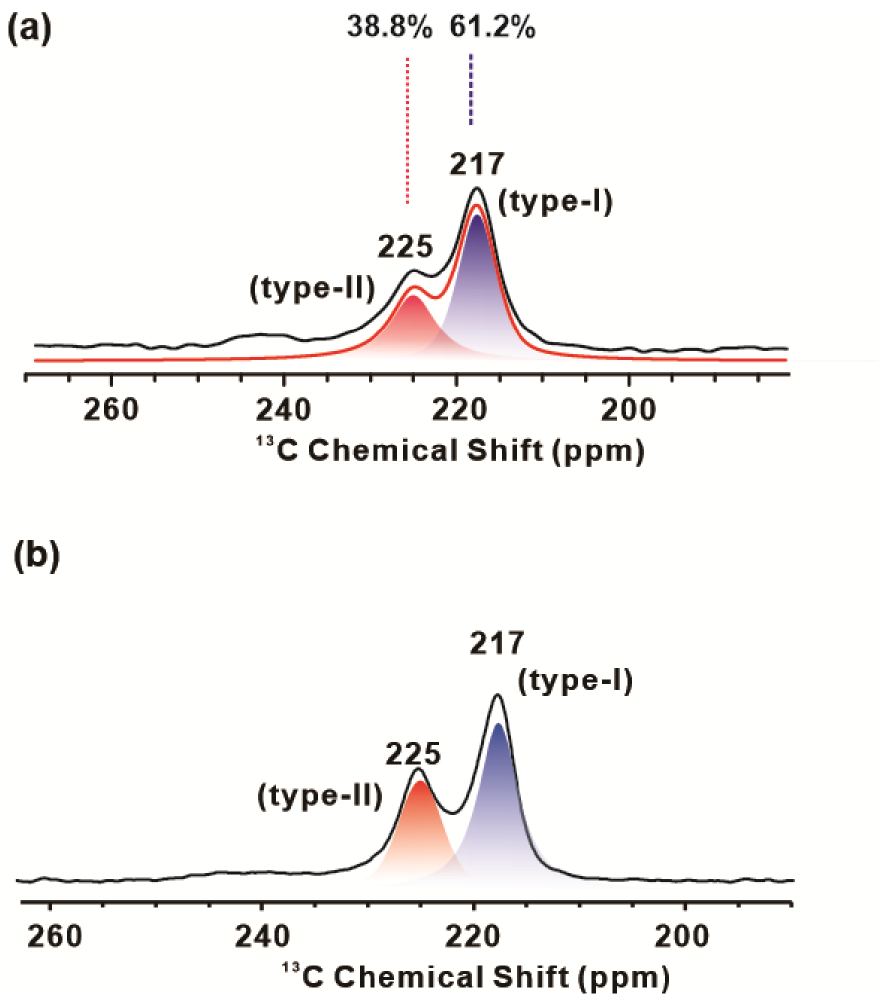

Figure S5. ^{13}C (a) one pulse and (b) cross polarization MAS SSNMR of SAPO-34 adsorbed with ^{13}C-2-acetone at 298 K. In (a) black and red curves represent the experimental and simulated spectra, respectively.

As shown in Figure S5, there are two types of acetone molecules with different adsorption modes (217 ppm and 225 ppm) in SAPO-34 zeolites at 298 K, which can be assigned to ^{13}C-2-acetone adsorbed on the BAS (Type-I) and the framework Al atoms (Type-II), respectively.

2.2 VT-SXRD results

VT-SXRD data of acetone-filled SAPO-34 were analyzed by TOPAS software using the crystallographic information file from Lo et al. (14) The background, scale and cell parameters were provisionally refined (a = 13.77(3), b = 13.77(3), c = 14.86(3)) to give a resulting Fourier map which was obtained using a step size of 0.2 Å.

According to our refinement procedure, acetone molecule was added into the SAPO-34 one by one (C1, C2, C3, ...). Fitting factors (Rwp / Rp / Rexp / $\chi 2$) were used to gauge the quality of the refinement. With the progressive increase in the number of acetone molecules, the R-factors were found decreasing, which indicates the use of higher number of acetones is approaching to the real structure (Table S2). It was until the third acetone, the R-factors were almost unchanged. However, the pseudo 'third' acetone appeared at the position very close to C2 (within 1.0 Å) which shared the site occupancy of C2. As provided in the SSNMR, there are two adsorption states of acetone. Thus, we excluded the C3 acetone to be a genuine position, which was possibly arisen owing to distortion/displacement of C2 due to thermal motion, etc. As a result, two acetone molecules were used to fit all the SXRD data accordingly.

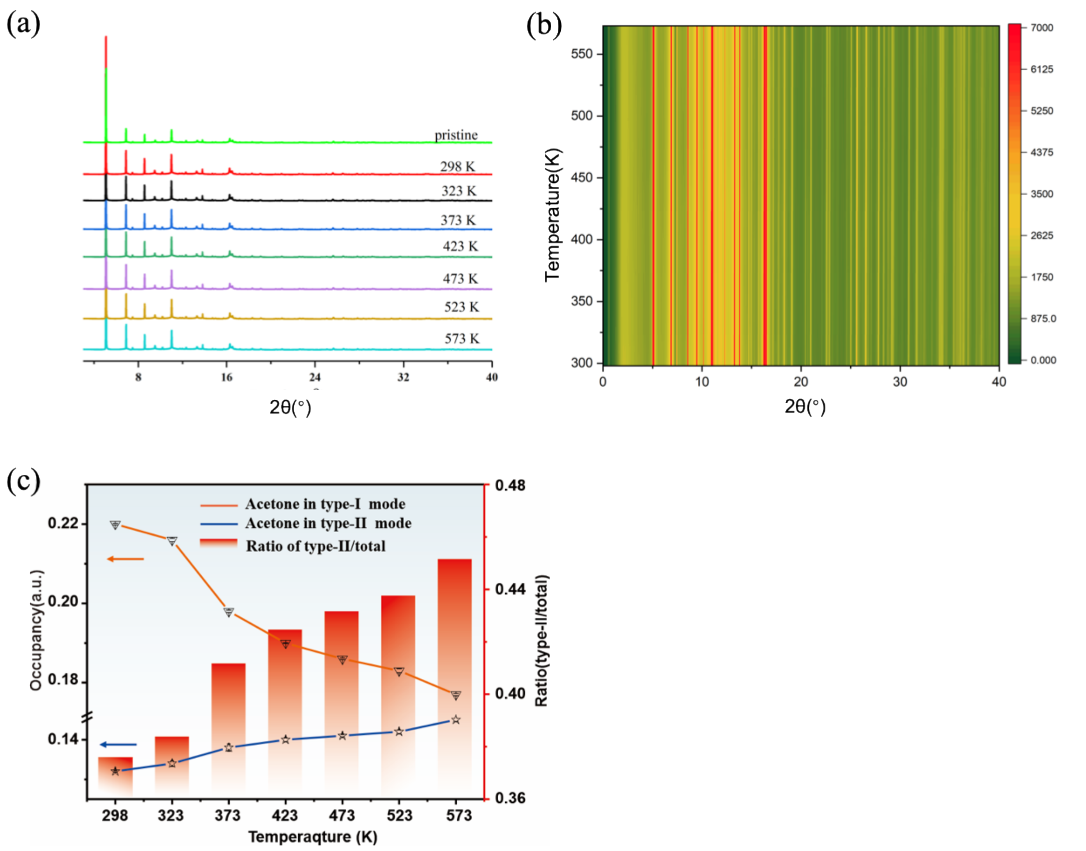

Figure S6. (a)1D and (b) 2D in situ VT-SXRD patterns of SAPO-34 before and after acetone adsorption at various temperatures,(c) the SOF of acetone in Type-I mode as a function of temperature in the range of 298 K to 573 K based on Table 1 by Reitveld refinement of VT-SXRD

Figure S6a displays the SXRD patterns of SAPO-34 zeolite before and after adsorption of acetone and scanned at different tempearture. it is found that the relative intensities of the peaks are changed upon addition of acetone, but without inducing new peaks or any significant peak broadening. This suggests that the acetone adsorption does not induce a major alteration to the parent SAPO-34 structure. Figure S6b shows the 2D SXRD patterns projection of SAPO-34 adsorbed with acetone as a function of temperature. Decreasing of peak intensity at high two theta angle is observed, indicting the thermal effect on the acetone adsorbed sample's structure. Figure S6c displays the SOF of acetone in Type-I mode as a function of temperature in the range of 298 K to 573 K. Despite the increasing thermal factors (greater errors in measurements as seen in increasing Beq values, see Tables S2-8), a notable progressive decrease in the SOF of acetone in the Type-I mode is observed, due to desorption and coversion to Type-II mode at elevated temperatures.

Table S1. Crystallographic data and details of acetone-adsorbed SAPO-34 collected at various temperatures.

Sample	Acetone@SAPO-34						
	298 K	323 K	373 K	423 K	473 K	523 K	573 K
**Crystal system**	Trigonal	Trigonal	Trigonal	Trigonal	Trigonal	Trigonal	Trigonal
**Space group**	R-3	R-3	R-3	R-3	R-3	R-3	R-3
**2θ range refinement**	3 – 45 º	3 – 45 º	3 – 45 º	3 – 45 º	3 – 45 º	3 – 45 º	3 – 45 º
**a (Å)**	13.76(4)	13.77(3)	13.74(2)	13.74(3)	13.74(2)	13.73(4)	13.73(2)
**b (Å)**	13.76(4)	13.77(2)	13.74(1)	13.74(1)	13.74(6)	13.73(3)	13.73(2)
**c (Å)**	14.94(3)	14.86(4)	14.86(3)	14.98(3)	14.98(3)	14.97(2)	14.96(6)
**V ($Å^3$)**	2448.17(35)	2439.10(18)	2452.12(24)	2451.32(20)	2448.82(25)	2446.72(15)	2444.43(20)
**Rwp / Rp / Rexp (%)**	8.5/6.3/1.7	8.9/6.8/4.5	8.7/6.7/1.5	8.9/6.8/1.6	9.7/6.9/1.6	8.4/6.1/1.6	9.8/6.9/1.6

2.2.1 The Rietveld refinement and details of acetone-adsorbed SAPO-34 at 298 K.

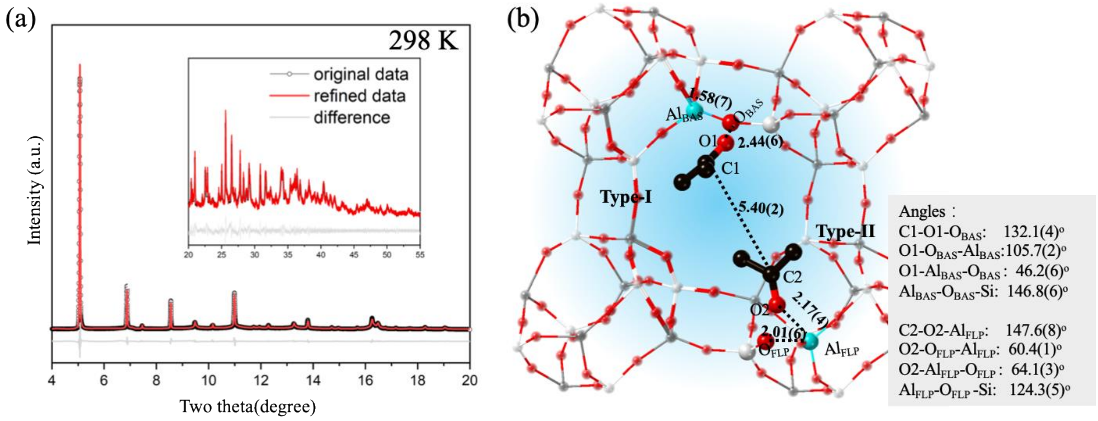

Figure S7. (a) SXRD refinement and (b) the structure of acetone-adsorbed SAPO-34 at 298 K. Comparison of the experimental data (black circle), the Rietveld refinement (red line), and the difference between them (grey line) for SXRD patterns.

Figure S7a shows the selected Rietveld refinement profiles of the SXRD patterns at 298 K; their corresponding derived crystal structures of acetone adsorption modes are plotted in Figures 2b and S7b; the refined structural parameters are also presented in Table S1. From the derived refinement data within acceptable errors, two adsorption configurations of acetone are clearly observed in the cage (Type-I: acetone on framework oxygen, Type-II: acetone on framework Al) at 298 K with the interatomic distance between C1 in Type-I acetone and C2 in Type-II acetone of 5.40(2) Å, which are in a good agreement with the PDSD MAS NMR results (Figure 1b). The ratio of the two adsorption modes from occupancy values presented in Table 1 also matches well with the ratio obtained from 1D one-pulse ^{13}C MAS NMR as shown in Figure S5. Thus, the structures and quantities of the two adsorbed acetone modes in the unit cell have been analyzed by both SSNMR and crystallographic methods with excellent agreement.

Furthermore, looking into the refined structure in detail (Figure S7), for acetone Type-I mode, the refined BAS (Al-O(H)-Si) bond angle and length (Al-O) are ca.146.8(6)° and 1.58(7) Å, respectively. The observed angle formed by the Type-I acetone oxygen (O_1), framework oxygen (O_{BAS}) and framework BAS aluminum (Al_{BAS}) ($\angle O_1$- O_{BAS}-Al_{BAS}) is ca.105.7(2)° which is significantly larger than the refined $\angle O_1$- Al_{BAS}- O_{BAS} at ca. 46.2(6)°, suggesting that acetone is adsorbed nearly atop to the O_{BAS} atom. Besides, bond distance of O_1-O_{BAS} is ca. 2.44(6) Å, although no H is refined by SXRD, this adsorption mode can be assigned to acetone adsorbed on BAS according to the literature.(15) As for the refined acetone Type-II mode, the adsorption geometry of C=O in acetone is clearly leaning toward the framework FLP Al (Al_{FLP}) with the $\angle O_2$- O_{FLP} - Al_{FLP} at ca. 60.4(1)° and the $\angle O_2$- Al_{FLP} - O_{FLP} at ca. 64.1(3)°, exhibiting

an interatomic distance between O of Type-II acetone (O_2) and Al_{FLP} at ca. 2.17(4) Å. The framework Al_{FLP} - O_{FLP} is apparently extended to ca. 2.01(6) Å which is significantly longer than that in the Type-I mode (A_{BAS} - O_{BAS}, ca. 1.58(7) Å), indicating the weakening of the framework Al_{FLP} − O_{FLP} interaction in the acetone Type-II mode. Those two different adsorption modes are also consistent with the information from ^{13}C–^{27}Al SRESPDOR experiment (Figure 1c).

Table S2. Crystallographic information files from the Rietveld refinement of acetone-adsorbed SAPO-34 at 298 K.

Species	Atom	X	Y	Z	SOF	Beq (Å2)
Zeolite framework	P1	0.4506(5)	0.1162(6)	1.4191(3)	0.84	0.18(11)
	P2	0.4240(5)	0.3351(9)	1.2166(6)	0.84	0.18(11)
	Si1	0.4506(5)	0.1162(6)	1.4191(3)	0.16	0.18(11)
	Si2	0.4240(5)	0.3351(9)	1.2166(6)	0.16	0.18(11)
	Al1	0.4431(1)	0.3302(3)	1.4286(1)	1	0.18(11)
	Al2	0.4296(1)	0.1049(3)	1.2125(2)	1	0.18(11)
	O1	0.4258(6)	0.0867(2)	1.3204(5)	1	0.18(11)
	O2	0.4136(1)	0.2074(1)	1.4554(7)	1	0.18(11)
	O5	0.4055(8)	0.3470(7)	1.3160(9)	1	0.18(11)
	O7	0.4514(0)	0.2342(3)	1.2047(2)	1	0.18(11)
	O3	0.5704(8)	0.1424(2)	1.4419(3)	0.5	0.18(11)
	O4a	0.3277(0)	0.0426(6)	1.1330(5)	0.5	0.18(11)
	O6	0.5404(5)	0.4402(9)	1.1958(7)	0.5	0.18(11)
	O8	0.3243(3)	0.3208(2)	1.1480(9)	0.5	0.18(11)
	O3a	0.5719(4)	0.4295(2)	1.4419(3)	0.5	0.18(11)
	O4	0.3816(3)	0.0056(5)	1.4663(8)	0.5	0.18(11)
	O6a	0.5597(1)	0.1001(6)	1.1958(7)	0.5	0.18(11)
	O8a	0.3458(5)	0.3368(4)	1.4814(2)	0.5	0.18(11)
Acetone (type-I)	C1	0.1755(2)	0.0599(5)	1.3521(6)	0.220(2)	6(3)
	C2	0.0878(3)	0.0438(2)	1.3983(4)	0.220(2)	6(3)
	C3	0.2069(0)	0.0543(2)	1.2563(1)	0.220(2)	6(3)
	O	0.2244(3)	0.1549(4)	1.3951(2)	0.220(2)	6(3)
Acetone (type-II)	C1a	0.6514(5)	0.4433(6)	1.0278(1)	0.132(2)	6(3)
	C2a	0.6652(3)	0.4080(4)	0.9353(3)	0.132(2)	6(3)
	C3a	0.7528(4)	0.5178(5)	1.0833(6)	0.132(2)	6(3)
	Oa	0.5516(4)	0.4094(3)	1.0597(2)	0.132(2)	6(3)

2.2.2 The Rietveld refinement and details of acetone-adsorbed SAPO-34 at 323 K.

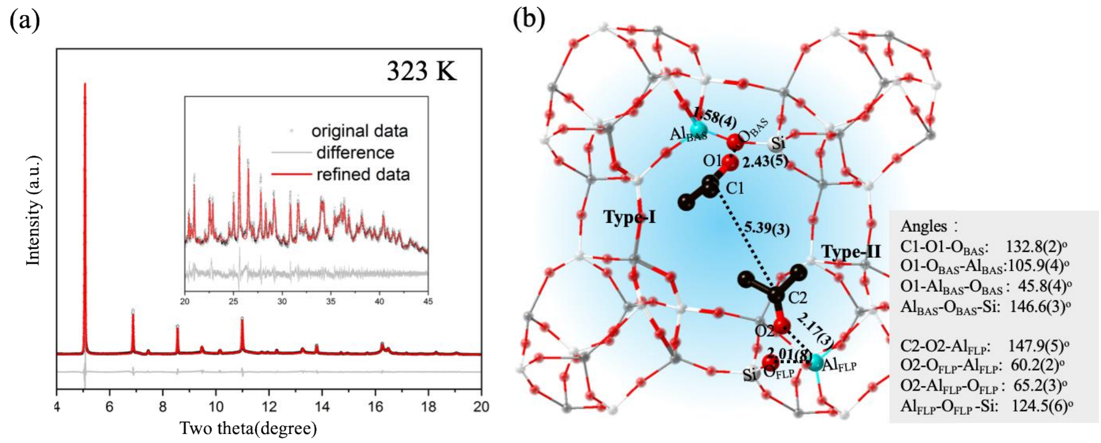

Figure S8. (a) SXRD refinement and (b) the structure of acetone-adsorbed SAPO-34 at 323 K. Comparison of the experiment data (black circle), the Rietveld refinement (red line), and the difference between them (grey line) for SXRD patterns.

Table S3. Crystallographic information files from the Rietveld refinement of acetone-adsorbed SAPO-34 at 323 K.

Species	Atom	X	Y	Z	SOF	Beq (Å2)
Zeolite framework	P1	0.4506(5)	0.1162(6)	1.4191(3)	0.84	0.18(11)
	P2	0.4240(5)	0.3351(9)	1.2166(6)	0.84	0.18(11)
	Si1	0.4506(5)	0.1162(6)	1.4191(3)	0.16	0.18(11)
	Si2	0.4240(5)	0.3351(9)	1.2166(6)	0.16	0.18(11)
	Al1	0.4431(1)	0.3302(3)	1.4286(1)	1	0.18(11)
	Al2	0.4296(1)	0.1049(3)	1.2125(2)	1	0.18(11)
	O1	0.4258(6)	0.0867(2)	1.3204(5)	1	0.18(11)
	O2	0.4136(1)	0.2074(1)	1.4554(7)	1	0.18(11)
	O5	0.4055(8)	0.3470(7)	1.3160(9)	1	0.18(11)
	O7	0.4514(0)	0.2342(3)	1.2047(2)	1	0.18(11)
	O3	0.5704(8)	0.1424(2)	1.4419(3)	0.5	0.18(11)
	O4a	0.3277(0)	0.0426(6)	1.1330(5)	0.5	0.18(11)
	O6	0.5404(5)	0.4402(9)	1.1958(7)	0.5	0.18(11)
	O8	0.3243(3)	0.3208(2)	1.1480(9)	0.5	0.18(11)
	O3a	0.5719(4)	0.4295(2)	1.4419(3)	0.5	0.18(11)
	O4	0.3816(3)	0.0056(2)	1.4663(8)	0.5	0.18(11)
	O6a	0.5597(1)	0.1001(6)	1.1958(7)	0.5	0.18(11)
	O8a	0.3458(5)	0.3368(4)	1.4814(2)	0.5	0.18(11)
Acetone(type-I)	C1	0.1755(6)	0.0599(7)	1.3521(6)	0.216(4)	6(3)
	C2	0.0878(3)	0.0439(8)	1.3983(7)	0.216(4)	6(3)
	C3	0.2069(0)	0.0543(5)	1.2563(9)	0.216(4)	6(3)
	O	0.2244(3)	0.1549(4)	1.3951(2)	0.216(4)	6(3)
Acetone(type-II)	C1a	0.6514(5)	0.4433(7)	1.0278(1)	0.134(7)	6(3)
	C2a	0.6652(3)	0.4080(4)	0.9353(9)	0.134(7)	6(3)
	C3a	0.7528(3)	0.5178(6)	1.0833(7)	0.134(7)	6(3)
	Oa	0.5516(4)	0.4094(3)	1.0597(6)	0.134(7)	6(3)

2.2.3 The Rietveld refinement and details of acetone-adsorbed SAPO-34 at 373 K.

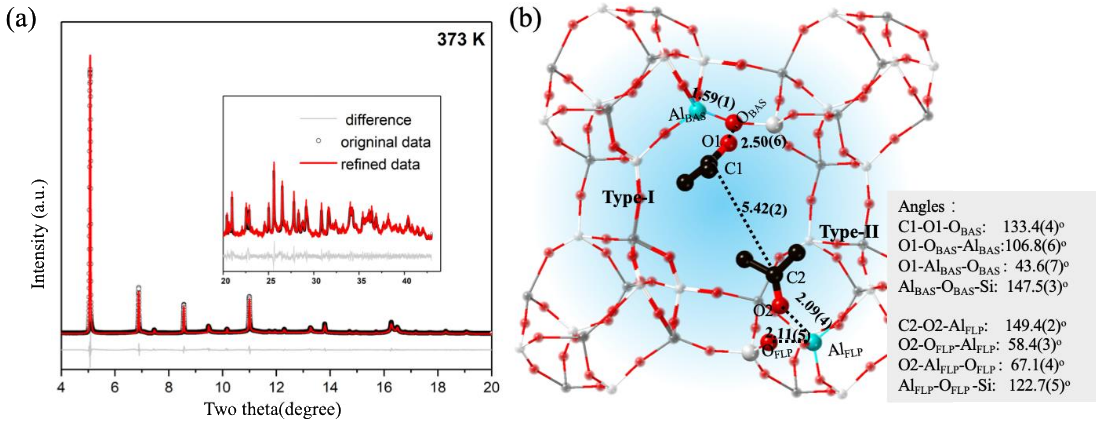

Figure S9. (a) SXRD refinement and (b)the structure of acetone-adsorbed SAPO-34 at 373 K. Comparison of the experiment data (black circle), the Rietveld refinement (red line), and the difference between them (grey line) for SXRD patterns.

Table S4. Crystallographic information files from the Rietveld refinement of acetone-adsorbed SAPO-34 at 373 K.

Species	Atom	X	Y	Z	SOF	Beq (Å2)
Zeolite framework	P1	0.4453(2)	0.1115(0)	1.4235(6)	0.84	0.32(30)
	P2	0.4392(2)	0.3336(8)	1.2183(2)	0.84	0.32(30)
	Si1	0.4453(2)	0.1115(0)	1.4235(6)	0.16	0.32(30)
	Si2	0.4392(2)	0.3336(8)	1.2183(2)	0.16	0.32(30)
	Al1	0.4361(2)	0.3329(5)	1.4279(2)	1	0.32(30)
	Al2	0.4329(2)	0.1067(4)	1.2123(5)	1	0.32(30)
	O1	0.4272(0)	0.0867(2)	1.3204(5)	1	0.63(50)
	O2	0.4110(4)	0.2060(4)	1.4538(5)	1	0.63(50)
	O5	0.4107(8)	0.3470(7)	1.3160(9)	1	0.63(50)
	O7	0.4686(6)	0.2342(3)	1.2047(2)	1	0.63(50)
	O3	0.5610(3)	0.1221(6)	1.4486(5)	0.5	0.63(50)
	O4a	0.5611(3)	0.0321(1)	1.1446(2)	0.5	0.63(50)
	O6	0.3755(5)	0.4325(4)	1.1810(7)	0.5	0.63(50)
	O8	0.5674(6)	0.3077(7)	1.1412(0)	0.5	0.63(50)
	O3a	0.3589(0)	0.4389(7)	1.4486(5)	0.5	0.63(50)
	O4	0.5611(3)	0.0101(0)	1.4779(5)	0.5	0.63(50)
	O6a	0.3755(5)	0.1052(0)	1.1810(7)	0.5	0.63(50)
	O8a	0.5674(6)	0.3633(2)	1.4745(3)	0.5	0.63(50)
Acetone (type-I)	C1	0.1755(6)	0.0599(7)	1.3521(6)	0.198(3)	8(2)
	C2	0.0878(3)	0.0439(8)	1.3983(7)	0.198(3)	8(2)
	C3	0.2069(0)	0.0543(5)	1.2563(9)	0.198(3)	8(2)
	O	0.2244(3)	0.1549(4)	1.3951(2)	0.198(3)	8(2)
Acetone (type-II)	C1a	0.6514(5)	0.4433(7)	1.0278(1)	0.138(9)	8(2)
	C2a	0.6652(3)	0.4080(4)	0.9353(9)	0.138(9)	8(2)
	C3a	0.7528(3)	0.5178(6)	1.0833(7)	0.138(9)	8(2)
	Oa	0.5516(4)	0.4094(3)	1.0597(6)	0.138(9)	8(2)

2.2.4 The Rietveld refinement and details of acetone-adsorbed SAPO-34 at 423 K.

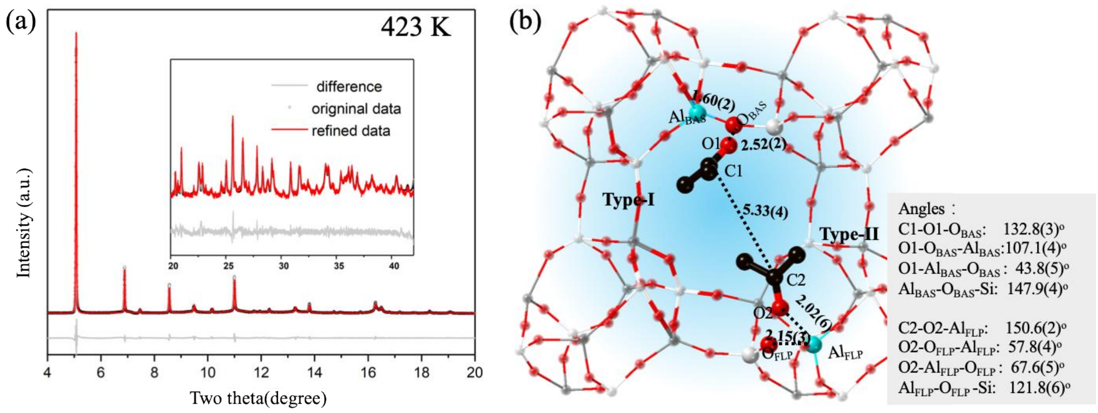

Figure S10. (a) SXRD refinement and (b)the structure of acetone adsorbed SAPO-34 at 423 K. Comparison of the experiment data (black circle), the Rietveld refinement (red line), and the difference between them (grey line) for SXRD patterns.

Table S5. Crystallographic information files from the Rietveld refinement of acetone-adsorbed SAPO-34 at 423 K.

Species	Atom	X	Y	Z	SOF	Beq (Å2)
Zeolite framework	P1	0.4506(5)	0.1162(6)	1.4191(3)	0.84	9.36(28)
	P2	0.4240(5)	0.3351(9)	1.2166(6)	0.84	9.36(28)
	Si1	0.4506(5)	0.1162(6)	1.4191(3)	0.16	9.36(28)
	Si2	0.4240(5)	0.3351(9)	1.2166(6)	0.16	9.36(28)
	Al1	0.4431(1)	0.3302(3)	1.4286(1)	1	9.36(28)
	Al2	0.4296(1)	0.1049(3)	1.2125(2)	1	9.36(28)
	O1	0.4258(6)	0.0867(2)	1.3204(5)	1	9.25(12)
	O2	0.4136(1)	0.2074(1)	1.4554(7)	1	9.25(12)
	O5	0.4055(8)	0.3470(7)	1.3160(9)	1	9.25(12)
	O7	0.451(4)	0.2342(3)	1.2047(2)	1	9.25(12)
	O3	0.5704(8)	0.1424(2)	1.4419(3)	0.5	9.25(12)
	O4a	0.327(7)	0.0426(6)	1.1330(5)	0.5	9.25(12)
	O6	0.5404(5)	0.4402(9)	1.1958(7)	0.5	9.25(12)
	O8	0.3243(3)	0.3208(2)	1.1480(9)	0.5	9.25(12)
	O3a	0.5719(4)	0.5719(4)	1.4419(3)	0.5	9.25(12)
	O4	0.3816(3)	0.3816(3)	1.4663(8)	0.5	9.25(12)
	O6a	0.5597(1)	0.5597(1)	1.1958(7)	0.5	9.25(12)
	O8a	0.3458(5)	0.3458(5)	1.4814(2)	0.5	9.25(12)
Acetone (type-I)	C1	0.1755(6)	0.0599(7)	1.3521(6)	0.190(1)	10(2)
	C2	0.0878(3)	-0.0439(8)	1.3983(7)	0.190(1)	10(2)
	C3	0.2069(0)	0.0543(5)	1.2563(9)	0.190(1)	10(2)
	O	0.2244(3)	0.1549(4)	1.3951(2)	0.190(1)	10(2)
Acetone (type-II)	C1a	0.6514(5)	0.4433(7)	1.0278(1)	0.140(5)	10(2)
	C2a	0.6652(3)	0.4080(4)	0.9353(9)	0.140(5)	10(2)
	C3a	0.7528(3)	0.5178(6)	1.0833(7)	0.140(5)	10(2)
	Oa	0.5516(4)	0.4094(3)	1.0597(6)	0.140(5)	10(2)

2.2.5 The Rietveld refinement and details of acetone-adsorbed SAPO-34 at 473 K.

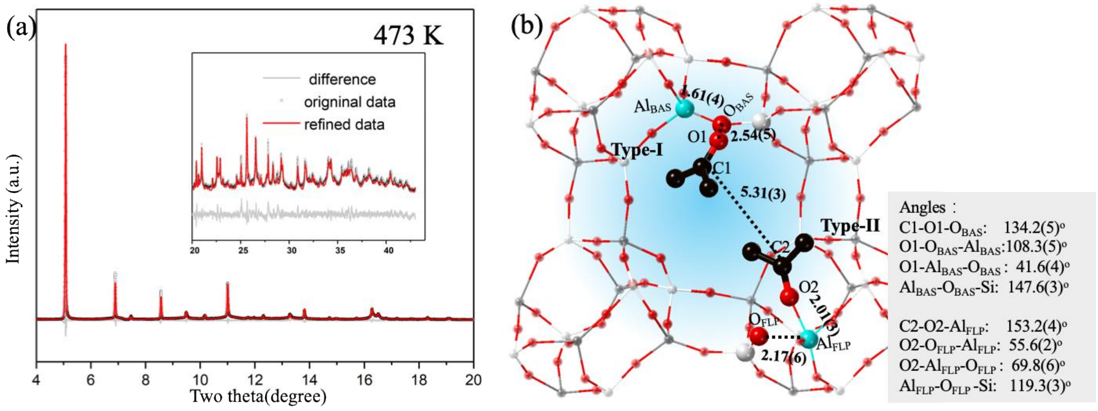

Figure S11. (a) SXRD refinement and (b) the structure of acetone-adsorbed SAPO-34 at 473 K. Comparison of the experiment data (black circle), the Rietveld refinement (red line), and the difference between them (grey line) for SXRD patterns.

Table S6. Crystallographic information files from the Rietveld refinement of acetone-adsorbed SAPO-34 at 473 K.

Species	Atom	X	Y	Z	SOF	Beq (Å2)
Zeolite framework	P1	0.4425(5)	0.1140(8)	1.4173(1)	0.84	15.196(12)
	P2	0.4262(2)	0.3365(2)	1.2131(3)	0.84	15.196(12)
	Si1	0.4425(5)	0.1140(8)	1.4173(1)	0.16	15.196(12)
	Si2	0.4262(2)	0.3365(2)	1.2131(3)	0.16	15.196(12)
	Al1	0.4397(6)	0.3428(7)	1.4275(7)	1	15.196(12)
	Al2	0.4255(8)	0.0989(1)	1.2076(8)	1	15.196(12)
	O1	0.4258(6)	0.0867(2)	1.3204(5)	1	15.670(27)
	O2	0.4136(1)	0.2074(1)	1.4554(7)	1	15.670(27)
	O5	0.4055(8)	0.3470(7)	1.3160(9)	1	15.670(27)
	O7	0.4514(4)	0.2342(3)	1.2047(2)	1	15.670(27)
	O3	0.5704(8)	0.1424(2)	1.4419(3)	0.5	15.670(27)
	O4a	0.3277(4)	0.0426(6)	1.1330(5)	0.5	15.670(27)
	O6	0.5404(5)	0.4402(9)	1.1958(7)	0.5	15.670(27)
	O8	0.3243(3)	0.3208(2)	1.1480(9)	0.5	15.670(27)
	O3a	0.5719(4)	0.1140(8)	1.4419(3)	0.5	15.670(27)
	O4	0.3816(3)	0.4295(2)	1.4663(8)	0.5	15.670(27)
	O6a	0.5597(1)	0.0056(3)	1.1958(7)	0.5	15.670(27)
	O8a	0.3458(5)	0.1001(6)	1.4814(2)	0.5	15.670(27)
Acetone(type-I)	C1	0.6430(5)	0.3368(4)	1.0982(4)	0.186(2)	10(2)
	C2	0.5556(7)	0.1452(9)	1.0696(7)	0.186(2)	10(2)
	C3	0.4474(5)	0.1439(8)	1.0526(2)	0.186(2)	10(2)
	O	0.5630(7)	0.0414(0)	1.0537(5)	0.186(2)	10(2)
Acetone(type-II)	C1a	0.1660(2)	0.1393(5)	1.2684(6)	0.141(3)	10(2)
	C2a	0.1258(3)	0.0184(3)	1.2472(1)	0.141(3)	10(2)
	C3a	0.1665(5)	0.1748(0)	1.3632(7)	0.141(3)	10(2)
	Oa	0.2003(1)	0.2134(2)	1.2047(1)	0.141(3)	10(2)

2.2.6 The Rietveld refinement and details of acetone-adsorbed SAPO-34 at 523 K.

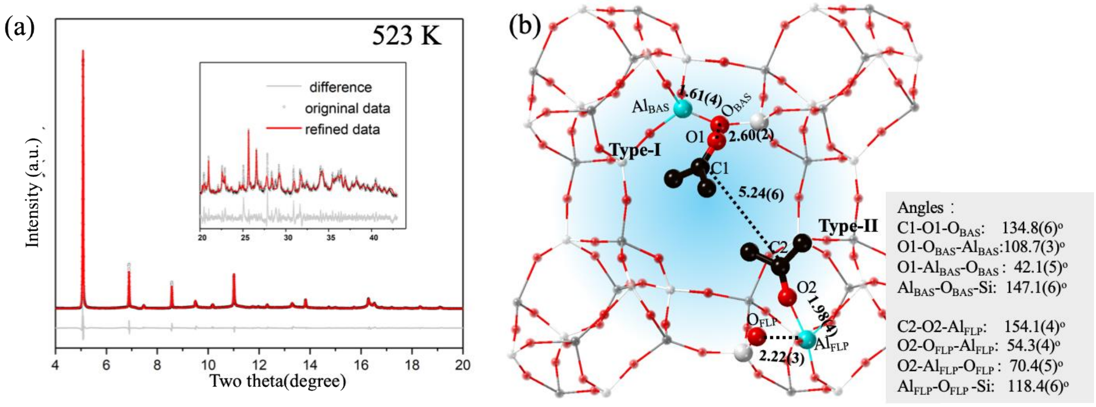

Figure S12. (a) SXRD refinement and (b) the structure of acetone-adsorbed SAPO-34 at 523 K. Comparison of the experiment data (black circle), the Rietveld refinement (red line), and the difference between them (grey line) for SXRD patterns.

Table S7. Crystallographic information files from the Rietveld refinement of acetone-adsorbed SAPO-34 at 523 K.

Species	Atom	X	Y	Z	SOF	Beq (Å2)
Zeolite framework	P1	0.4584(9)	0.1446(6)	1.4031(8)	0.84	16.75(57)
	P2	0.4362(2)	0.3232(9)	1.2421(4)	0.84	16.75(57)
	Si1	0.4584(9)	0.1446(3)	1.4031(8)	0.16	16.75(57)
	Si2	0.4362(2)	0.3232(5)	1.2421(4)	0.16	16.75(57)
	Al1	0.4444(0)	0.3204(7)	1.4078(4)	1	16.75(57)
	Al2	0.4134(5)	0.1016(8)	1.2279(5)	1	16.75(57)
	O1	0.4258(6)	0.0867(2)	1.3204(5)	1	19.88(20)
	O2	0.4136(1)	0.2074(1)	1.4554(7)	1	19.88(20)
	O5	0.4055(8)	0.3470(7)	1.3160(9)	1	19.88(20)
	O7	0.4514(0)	0.2342(3)	1.2047(2)	1	19.88(20)
	O3	0.5704(8)	0.1424(2)	1.4419(3)	0.5	19.88(20)
	O4a	0.3277(0)	0.0426(6)	1.1330(5)	0.5	19.88(20)
	O6	0.5404(5)	0.4402(9)	1.1958(7)	0.5	19.88(20)
	O8	0.3243(3)	0.3208(2)	1.1480(9)	0.5	19.88(20)
	O3a	0.5719(4)	0.4295(2)	1.4419(3)	0.5	19.88(20)
	O4	0.3816(3)	0.0056(3)	1.4663(8)	0.5	19.88(20)
	O6a	0.5597(1)	0.1001(6)	1.1958(7)	0.5	19.88(20)
	O8a	0.3458(6)	0.3368(4)	1.4814(2)	0.5	19.88(20)
Acetone (type-I)	C1	0.6430(8)	0.2364(9)	1.0982(5)	0.183(5)	20(2)
	C2	0.5556(7)	0.1452(9)	1.0696(7)	0.183(5)	20(2)
	C3	0.4474(2)	0.1439(8)	1.0526(2)	0.183(5)	20(2)
	O	0.5630(7)	0.0413(7)	1.0537(5)	0.183(5)	20(2)
Acetone (type-II)	C1a	0.1660(1)	0.1393(5)	1.2684(6)	0.42(5)	20(2)
	C2a	0.1258(7)	0.0183(9)	1.2472(0)	0.42(5)	20(2)
	C3a	0.1665(4)	0.1748(1)	1.3633(0)	0.42(5)	20(2)
	Oa	0.2003(3)	0.2134(4)	1.2046(9)	0.42(5)	20(2)

2.2.7 The Rietveld refinement and details of acetone adsorbed SAPO-34 at 573 K.

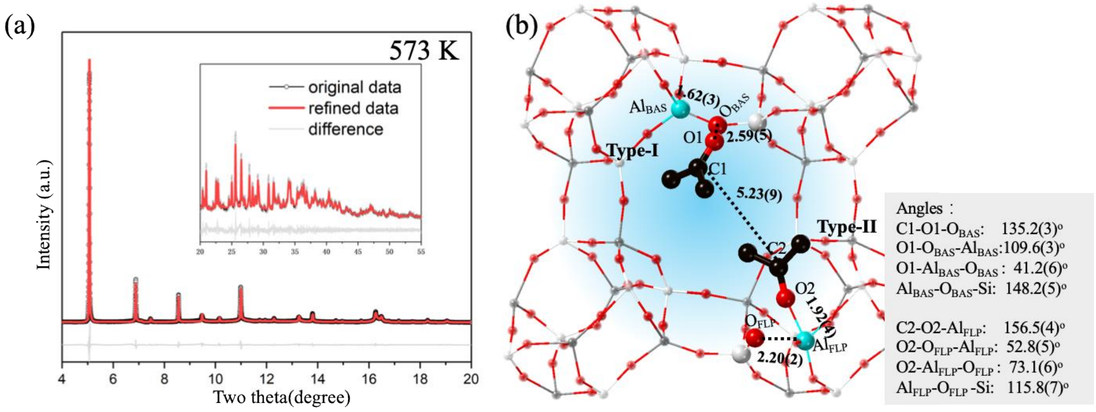

Figure S13. (a) SXRD refinement and (b) the structure of acetone adsorbed SAPO-34 at 573 K. Comparison of the experiment data (black circle), the Rietveld refinement (red line), and the difference between them (grey line) for SXRD patterns.

Table S8. Crystallographic information files from the Rietveld refinement of acetone-adsorbed SAPO-34 at 573 K.

Species	Atom	X	Y	Z	SOF	Beq (Å2)
Zeolite framework	P1	0.4594(7)	0.1410(4)	1.4053(3)	0.84	16.75(57)
	P2	0.4394(6)	0.3284(2)	1.2354(3)	0.84	16.75(57)
	Si1	0.4594(7)	0.1410(4)	1.4053(1)	0.16	16.75(57)
	Si2	0.4394(6)	0.3284(2)	1.2354(3)	0.16	16.75(57)
	Al1	0.4457(3)	0.3215(1)	1.4108(2)	1	16.75(57)
	Al2	0.4167(9)	0.1009(0)	1.2257(3)	1	16.75(57)
	O1	0.4258(6)	0.0867(2)	1.3204(5)	1	19.88(20)
	O2	0.4136(1)	0.2074(1)	1.4554(7)	1	19.88(20)
	O5	0.4055(8)	0.3470(7)	1.3160(9)	1	19.88(20)
	O7	0.4514(3)	0.2342(3)	1.2047(2)	1	19.88(20)
	O3	0.5704(8)	0.1424(2)	1.4419(3)	0.5	19.88(20)
	O4a	0.3277(5)	0.0426(6)	1.1330(5)	0.5	19.88(20)
	O6	0.5404(5)	0.4402(9)	1.1958(7)	0.5	19.88(20)
	O8	0.3243(3)	0.3208(2)	1.1480(9)	0.5	19.88(20)
	O3a	0.5719(4)	0.4295(2)	1.4419(3)	0.5	19.88(20)
	O4	0.3816(3)	0.0056(3)	1.4663(8)	0.5	19.88(20)
	O6a	0.5597(1)	0.1001(6)	1.1958(7)	0.5	19.88(20)
	O8a	0.3458(5)	0.3368(4)	1.4814(2)	0.5	19.88(20)
Acetone (type-I)	C1	0.4229(5)	0.1446(0)	1.2684(6)	0.177(3)	20(2)
	C2	0.4548(0)	0.2290(8)	0.9825(1)	0.177(3)	20(2)
	C3	0.4015(5)	0.3013(0)	1.0368(0)	0.177(3)	20(2)
	O	0.5447(8)	0.2543(2)	1.0315(9)	0.177(3)	20(2)
Acetone (type-II)	C1a	0.1660(1)	0.1393(5)	1.1046(5)	0.145(4)	20(2)
	C2a	0.1258(6)	0.0183(6)	1.2471(9)	0.145(4)	20(2)
	C3a	0.1665(4)	0.1748(2)	1.3633(4)	0.145(4)	20(2)
	Oa	0.2003(4)	0.2134(6)	1.2046(6)	0.145(4)	20(2)

2.3 In situ DRIFTS results

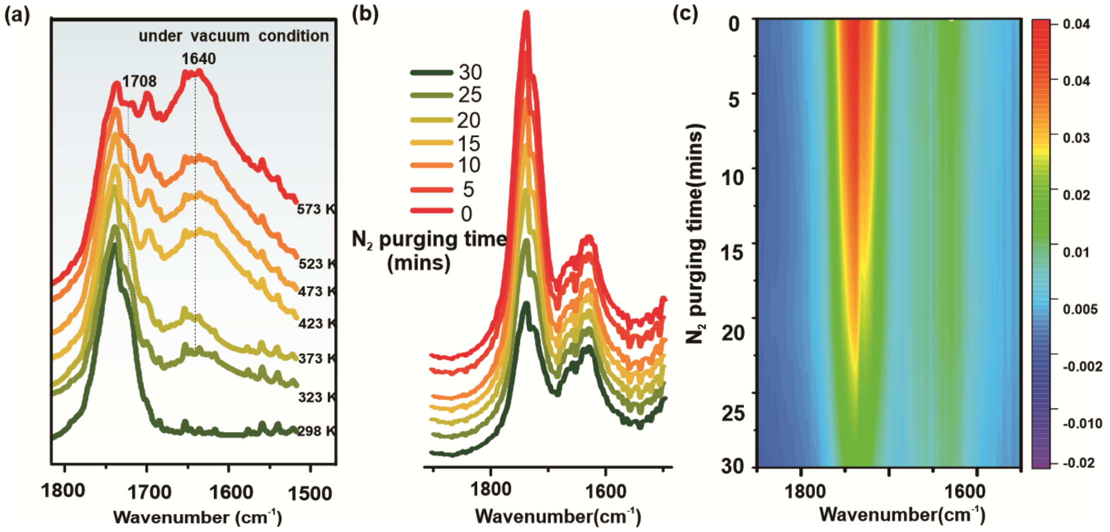

Figure S14. (a) In-situ DRIFT spectra of acetone adsorbed on SAPO-34 heated from 298 K to 573 K under vacuum condition, (b) 1D and (c) 2D DRIFT spectra of the acetone-adsorbed SAPO-34 as a function of N_2 purging time recorded at 573 K.

Figure S14a shows the in-situ DRIFT results of acetone adsorbed over SAPO-34 under vacuum condition. Although the gas peak of acetone cannot be totally removed by the dyamic vacuum, the band at 1708 cm^{-1} (Type-I) is much clearer under room temperature and band of acetone in Type-II mode (1640 cm^{-1}) is also increased and becomes significant at elevated temperatures. However, it should be cautious that the inter-conversion of Type-I to Type-II under vacuum is expected to be very different from the flow conditions due to their differences of their surface lifetimes and desorption values. For example, AIMD result (Figure 3d) suggests that the physical adsorption of acetone works only as a transition state during the conversion, which may be affected during the conversion under vacuum. Figure S14b displays DRIFT spectra peak change of SAPO-34 zeolite adsorbed with acetone under 573 K as a function of N_2 purging time, the corresponding 2D peak intensity projection is shown in Figure S14c. It is clear to see that the band at ca.1640 cm^{-1} remain almost unchanged whereas those bands ranging between 1700 and 1740 cm^{-1} decrease gradually upon prolonging the purging time.

2.4 AIMD results

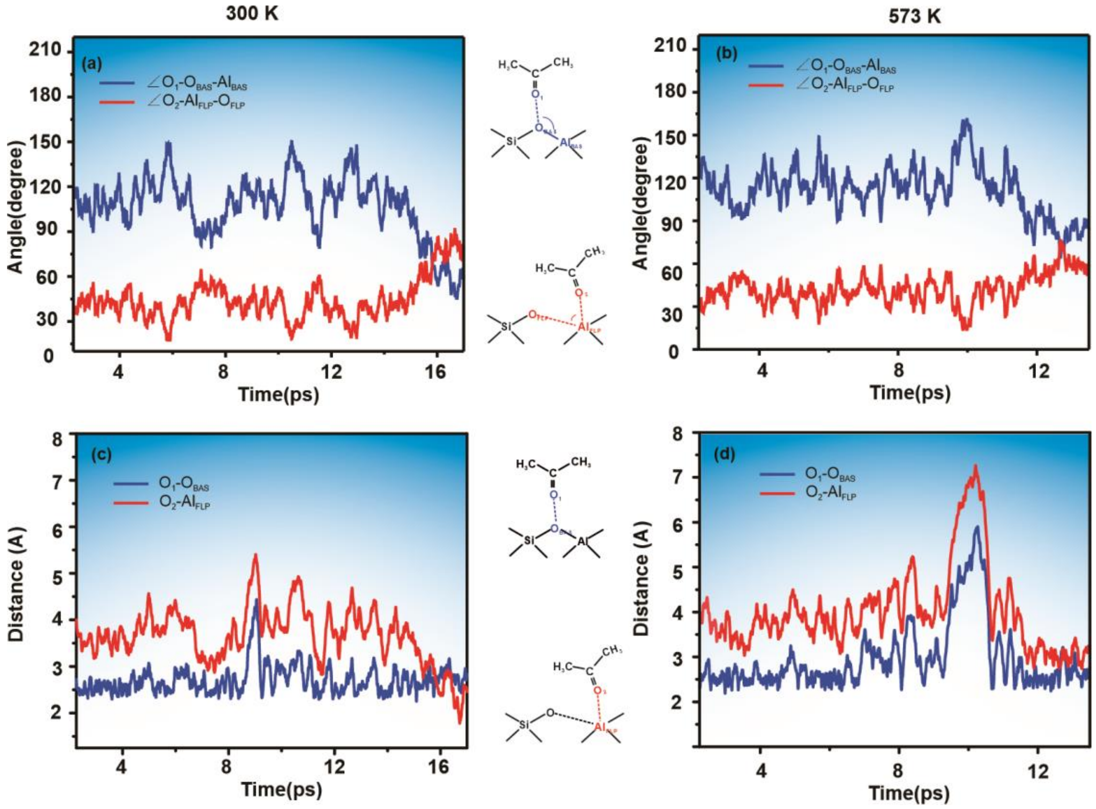

Figure S15. The changes of bond angles (O_1 -O_{BAS} -Al_{BAS} in blue, O_2 -Al_{FLP} -O_{FLP} in red) and distances (O_1 -O_{BAS} in blue, O_2 -Al_{FLP} in red) for acetone adsorbed on H-SAPO-34 with the dynamic transformation from initial BAS adsorption state to induced FLP adsorption state during the AIMD simulations at 300 K ((a)for angles, (c)for distances) and 573 K((b)for angles, (d)for distances). It is noted that the changes in angle and bond distance start at 15ps at 300K but 10ps at 573K together with the desorption of acetone species.

2.5 Movies

Movie S1: Episode of AIMD simulation for the transformation of acetone in Type-I to Type-II over SAPO-34 under 300 K.

Movie S2: Episode of AIMD simulation for the transformation of acetone in Type-I to Type-II over SAPO-34 under 573 K.

3 References

[1] Fernandez, Christian, J. P. Amoureux. "2D multiquantum MAS-NMR spectroscopy of ^{27}Al in aluminophosphate molecular sieves." *Chem. Phys. Let.* **242**. 449 (1995).

[2] G. Li, C. Foo, X. Yi, et al. Induced Active Sites by Adsorbate in Zeotype Materials. *J. Am. Chem. Soc.* **143**, 8761 (2021).

[3] J. P. Perdew, K. Burke, M. J. P. r. l. Ernzerhof, Generalized gradient approximation made simple. *Phys. Rev. Lett.* **77**, 3865 (1996).

[4] S. Grimme, J. Antony, S. Ehrlich, et al, A consistent and accurate ab initio parametrization of density functional dispersion correction (DFT-D) for the 94 elements H-Pu. *J. Chem. Phys.* **132**, 154104 (2010).

[5] G. Lippert, J. Hutter, M. Parrinello, The Gaussian and augmented-plane-wave density functional method for ab initio molecular dynamics simulations. *Theor. Chem. Acc.* **103**, 124 (1999).

[6] S. Goedecker, M. Teter, J. Hutter, Separable dual-space Gaussian pseudopotentials. *Phys. Rev. B* **54**, 1703 (1996).

[7] H. A. Posch, W. G. Hoover, F. J. Vesely, Canonical dynamics of the Nosé oscillator: stability, order, and chaos. *Phys. Rev. A* **33**, 4253 (1986).

[8] A. Laio, M. Parrinello, Escaping free-energy minima. *PNAS* **99**, 12562 (2002).

[9] L. Liu, B. Lukose, P. Jaque, et al. Reaction mechanism of hydrogen activation by frustrated Lewis pairs. *Green Energy Environ.* **4**, 20 (2019).

[10] A.Omegna, J. A. van Bokhoven, R.Prins, Flexible aluminum coordination in alumino-silicates. Structure of zeolite H-USY and amorphous silica-alumina. *J. Phys. Chem. B* **107**, 8854 (2003).

[11] A. Buchholz, W. Wang, M. Xu, et al. Thermal stability and dehydroxylation of Brønsted acid sites in silicoaluminophosphates H-SAPO-11, H-SAPO-18, H-SAPO-31, and H-SAPO-34 investigated by multi-nuclear solid-state NMR spectroscopy. *Micropor. Mesopor. Mat.* **56**, 267(2002).

[12] Z. Yu, A. Zheng, Q. Wang, et al. Insights into the. dealumination of zeolite HY revealed by sensitivity‐enhanced ^{27}Al DQ‐MAS NMR spectroscopy at high field. *Angew. Chem. Int. Ed.* **122**, 8839 (2010).

[13] R.Vomscheid, M. Briend, M. J. Peltre, et al. The role of the template in directing the Si distribution in SAPO zeolites. *J. Phys. Chem.* **98**, 9614 (1994).

[14] B. T. W. Lo, L.Ye, G. Chang, et al. Dynamic modification of pore opening of SAPO-34 by adsorbed surface methoxy species during induction of catalytic methanol-to-olefins reactions. *Appl. Catal. B.* **237**, 245 (2018).

[15] B. T. W. Lo, L.Ye, J. Qu, et al. Elucidation of Adsorbate Structures and Interactions on Brønsted Acid Sites in H-ZSM-5 by Synchrotron X-ray Powder Diffraction. *Angew. Chem. Int. Ed.* **128**, 6085 (2016).
